# Linking Inflammation, Aberrant Glutamate-Dopamine Interaction, and Post-synaptic Changes: Translational Relevance for Schizophrenia and Antipsychotic Treatment: a Systematic Review

**DOI:** 10.1007/s12035-022-02976-3

**Published:** 2022-08-13

**Authors:** Andrea de Bartolomeis, Annarita Barone, Licia Vellucci, Benedetta Mazza, Mark C. Austin, Felice Iasevoli, Mariateresa Ciccarelli

**Affiliations:** 1 Laboratory of Molecular and Translational Psychiatry, University School of Medicine of Naples Federico II, Naples, Italy; 2 Unit of Treatment Resistant Psychosis, Section of Psychiatry, Department of Neuroscience, Reproductive Science and Odontostomatology, University School of Medicine of Naples Federico II, Naples, Italy; 3 Clinical Psychopharmacology Program, College of Pharmacy, Idaho State University (ISU), Pocatello, ID USA

**Keywords:** Inflammation, Microglia, Post-synaptic density, Treatment-resistant schizophrenia, Interleukin, Clozapine

## Abstract

Evidence from clinical, preclinical, and post-mortem studies supports the inflammatory/immune hypothesis of schizophrenia pathogenesis. Less evident is the link between the inflammatory background and two well-recognized functional and structural findings of schizophrenia pathophysiology: the dopamine-glutamate aberrant interaction and the alteration of dendritic spines architecture, both believed to be the “quantal” elements of cortical-subcortical dysfunctional network. In this systematic review, we tried to capture the major findings linking inflammation, aberrant glutamate-dopamine interaction, and post-synaptic changes under a direct and inverse translational perspective, a paramount picture that at present is lacking. The inflammatory effects on dopaminergic function appear to be bidirectional: the inflammation influences dopamine release, and dopamine acts as a regulator of discrete inflammatory processes involved in schizophrenia such as dysregulated interleukin and kynurenine pathways. Furthermore, the link between inflammation and glutamate is strongly supported by clinical studies aimed at exploring overactive microglia in schizophrenia patients and maternal immune activation models, indicating impaired glutamate regulation and reduced N-methyl-D-aspartate receptor (NMDAR) function. In addition, an inflammatory/immune-induced alteration of post-synaptic density scaffold proteins, crucial for downstream NMDAR signaling and synaptic efficacy, has been demonstrated. According to these findings, a significant increase in plasma inflammatory markers has been found in schizophrenia patients compared to healthy controls, associated with reduced cortical integrity and functional connectivity, relevant to the cognitive deficit of schizophrenia. Finally, the link between altered inflammatory/immune responses raises relevant questions regarding potential new therapeutic strategies specifically for those forms of schizophrenia that are resistant to canonical antipsychotics or unresponsive to clozapine.

## Introduction

Schizophrenia is conceptualized as a neurodevelopmental disorder, putatively characterized at the molecular level by aberrant synaptic plasticity [1] and disorganized cortical-subcortical connectivity [2]. Multiple preclinical [3] and clinical findings converge to the possibility that inflammation may significantly impact the underlying neurobiology of the disease, contributing to abnormalities of neuronal signaling, synapse organization, and brain connectivity [4–7]. The contribution of inflammation to the development of schizophrenia should be interpreted in the framework of few basic facts related to the disease and reported robustly over time: the neurodevelopmental onset of schizophrenia; the consistently replicated association between major histocompatibility complex (MHC) region and Complement Component 4 (*C4*) genes and schizophrenia [8–11]; the possibility that schizophrenia could be viewed as a systemic disorder; the reproducibility of the profound, albeit subtle, alterations in microglia and dendritic spine architecture in animal models of early life inflammation; the imbalance of multiple neurotransmitter systems. Among others, the dopamine-glutamate interplay is believed to be the main disrupted synaptic and intracellular signaling in schizophrenia, according to in vivo PET studies [12], in vitro and in vivo animal modeling [13], as well as post-mortem brain tissue analysis [14–18]. On the other hand, psychotic symptoms reminiscent of schizophrenia can occur during infections of the central nervous system (CNS) [19] and autoimmune disorders [20, 21] pointing to a reciprocal interaction between neurotransmitter systems and immune mediators. Therefore, a bidirectional relationship between dopamine (and/or glutamate) dysfunctions and inflammation has recently emerged [22]. Furthermore, treatment-resistant schizophrenia (TRS), characterized by severe cognitive impairment, prominent structural and functional brain abnormalities, and poor prognosis, has been associated with peculiar immune signatures and deregulated inflammatory responses [23–25].

Despite the relevance of the issue and the increasing literature, no systematic review has been published on the topic; therefore, we aimed at tackling this issue by trying to answer the following questions:

1. How does the inflammation hypothesis of schizophrenia and recent findings on inflammatory response in schizophrenia patients fit in the framework of dopamine-glutamate altered interaction and vice versa?
2. How and to what extent do inflammation and related immune system alterations affect the function and structure of the synapse and are coherent with the changes of the dendritic spines architecture described in schizophrenia?
3. What is the evidence of genetic contribution to inflammation in schizophrenia pathophysiology?
4. How do the available antipsychotic treatments impact the putative effects of inflammation in schizophrenia?
5. What is the role of inflammation and the immune system in those forms of schizophrenia that do not respond to antipsychotics and what is the next scenario of therapy putatively based on anti-inflammatory effects?

## Material and Methods

The aim of the present systematic review is to provide an updated overview of the available evidence on the link between inflammation and aberrations of synaptic plasticity in schizophrenia, discussing its relevance for understanding the pathophysiology and developing novel therapeutic antipsychotic strategies.

**Fig. 1:**
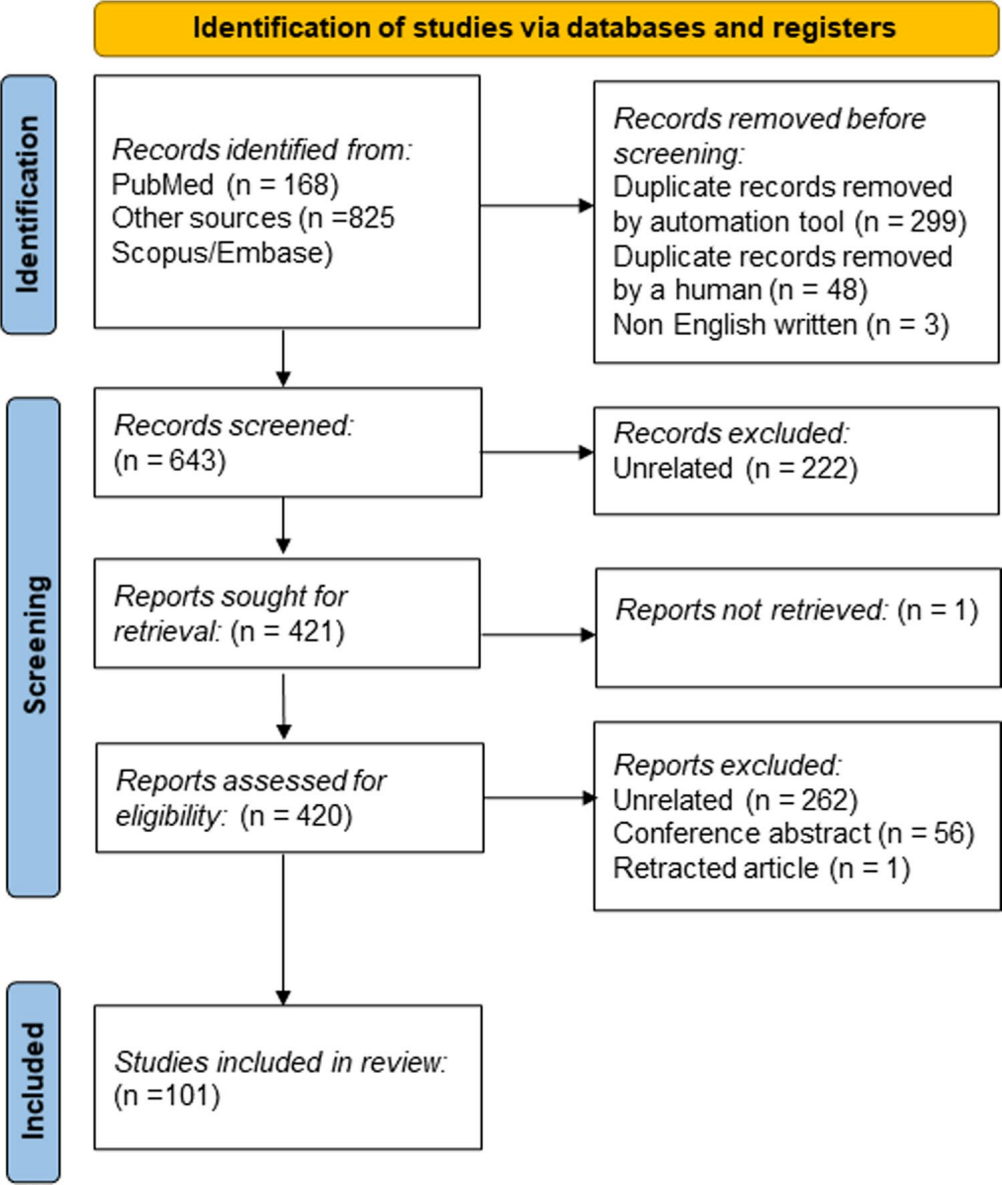
PRISMA flow diagram showing the flow of information through the different phases of the systematic review

The search and selection process has been conducted according to the PRISMA guidelines in order to identify eligible clinical and preclinical studies investigating the reciprocal relationship between immune-inflammatory dysregulations and synaptic disruption in schizophrenia. Therefore, the following searches were carried out on EMBASE, Scopus, and PubMed on 8th November 2021 (the last interrogation was conducted on 13th April 2022): ((schizophrenia) AND (dopamine-glutamate interaction)) AND (synap*); ((schizophrenia) AND (inflammation)) AND (synap*); (((schizophrenia) AND (inflammation)) AND (antipsychotic*)) AND (synap*), ((((dopamin*) AND (glutamat*)) AND (synap*)) AND (schizophrenia)) AND (infammation); (((((schizophrenia) AND (dopamin*)) AND (glutamat*)) AND (synap*)) AND (inflammation)) AND (antipsychotic*). The PRISMA flow diagram has been reported in Fig. 1. We deemed eligible English-written articles, published in peer-reviewed journals, exploring the link between inflammatory/immune processes and synaptic abnormalities. No time constraints were applied, and only original clinical and preclinical research studies and reviews were included. Conference abstracts, and commentaries were excluded. The search returned a total of 993 articles. One hundred and one articles were included in the qualitative synthesis and divided into topics of interest corresponding to the following sections.

## Early Life Infections/Inflammation in Schizophrenia

Early life adverse events, including maternal infections and perinatal stress, have been shown to alter neurodevelopmental processes and favor susceptibility to schizophrenia later in life [26, 27] Accordingly, epidemiological research has demonstrated significant associations between a wide range of early life risk factors and schizophrenia [28], including prenatal infections, maternal inflammation during pregnancy, obstetric complications, and neonatal and childhood infections [27, 29–31].

**Fig. 2:**
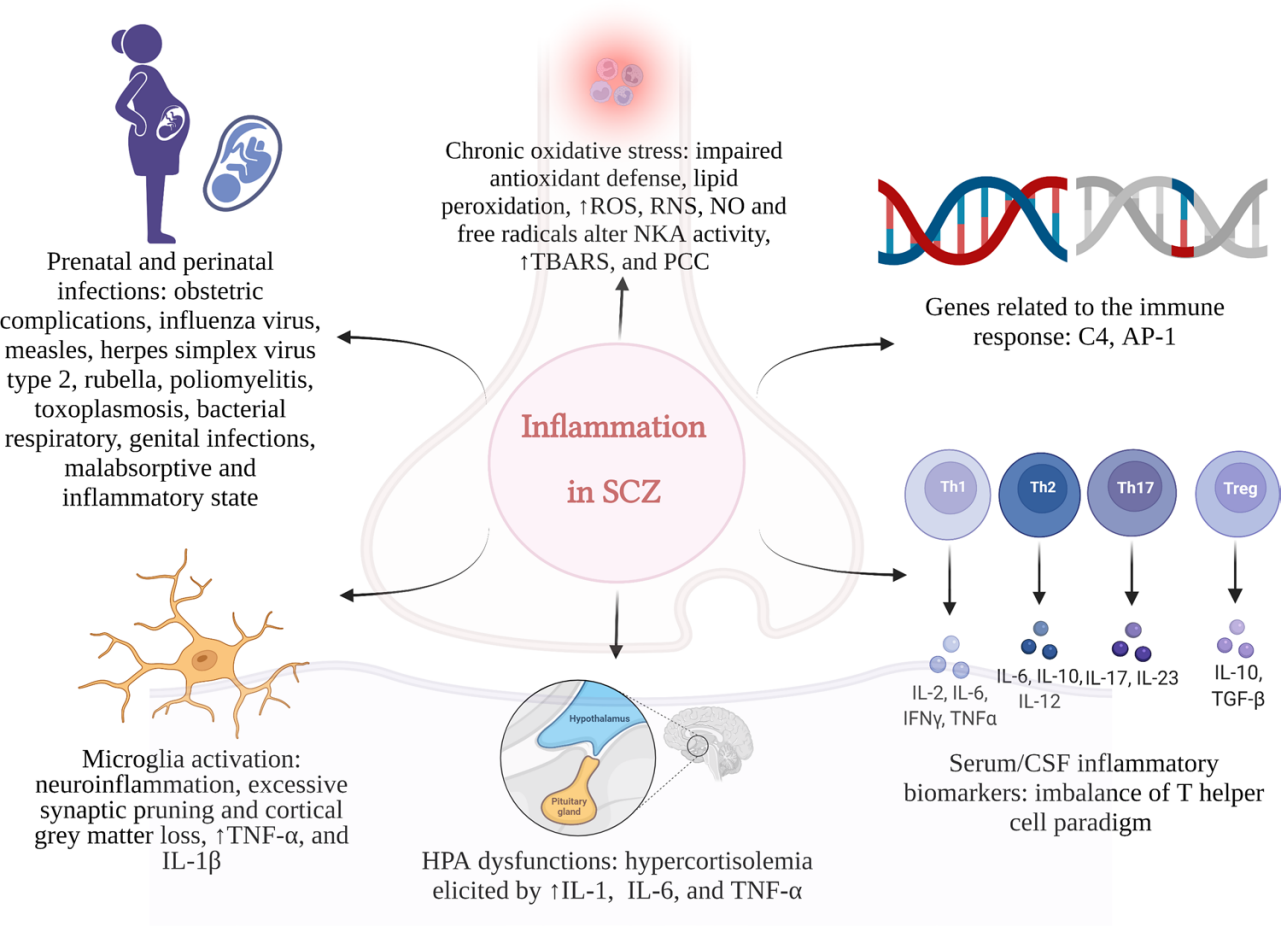
Potential role of inflammation in schizophrenia pathogenesis: Inflammation-induced immune alteration may represent a common pathway for environmental and genetic risk factors in schizophrenia, resulting in aberration of synaptic plasticity. SCZ (schizophrenia); ROS (reactive oxidative species); RNS (nitrosative species); NO (nitric oxide); NKA (Na + /K + -ATPase); TBARS (thiobarbituric acid-reactive substances); PCC (protein carbonyl content); C4 (complement component 4); AP-1 (activator protein 1); TNF-α (tumor necrosis factor α); IL-1β (interleukin 1β); IL-1 (interleukin 1); IL-6 (interleukin 6); CFS (cerebrospinal fluid); IL-2(interleukin 2); IFN-γ (interferon γ); IL-10 (interleukin 10); IL-12 (interleukin 12); IL-17 (interleukin 17); IL-10 (interleukin 10); TGF-β (transforming growth factor β). Created with BioRender.com

Since the 1918 influenza pandemic was followed by multiple reports of post-influenza psychoses and schizophrenia-like symptoms [32–34], it has been suggested that several bacterial and viral infections may be causally related to psychosis. In particular, influenza, measles, herpes simplex virus type 2, rubella, poliomyelitis, toxoplasmosis, and bacterial respiratory and genital infections gained the strongest associations with the disease (Fig. 2) [35–37].

Animal models further confirmed the associations between maternal immune activation (MIA) and behavioral disorders in the progeny. MIA models involving placental dysfunction and disruption of cytokine network culminate in the activation of microglia, influencing the number, structure, positioning, and survival of glial cells, contributing to morphological and functional changes in the brain [38, 39]. In fact, prenatal exposure to inflammation results in altered gene expression profile in hippocampal structures and widespread changes in γ-aminobutyric acid (GABA)ergic, glutamatergic, and serotonergic neuronal circuits mimicking those observed in schizophrenia, thus providing the basis for a model of infection-induced psychosis [27, 40–42].

Finally, inadequate maternal diet could also play a role by inducing a malabsorptive and inflammatory state that may potentially disturb pregnancy and fetal development, predisposing to neurodevelopmental disorders including schizophrenia [43].

Taken together, these works support an emerging role for the inflammation in the pathogenesis of schizophrenia. The specific contribution mediated by the inflammation on the molecular features of the disease will be detailed in the following sections (Fig. 2) [44].

## Immune Response in Schizophrenia

### Central and Peripheral Cytokines in Schizophrenia

It has been proposed that immunological responses, including innate and adaptive immunity, may mediate pathophysiological processes responsible for the onset of schizophrenia [45].

During MIA, pro-inflammatory mediators such as cytokines, chemokines, antibodies, and acute phase proteins are released into the maternal bloodstream, increasing the permeability of the placental barrier and the fetal blood–brain barrier, allowing inflammatory mediators to enter the fetal brain. In the CNS, these pro-inflammatory mediators can activate microglial cells that can release, in turn, pro-inflammatory cytokines such as TNF-α (tumor necrosis factor), IL-1β, and IL-6. As a result, circulating immune cells can infiltrate the brain, increasing cytokine levels and releasing antibodies that exacerbate inflammation by affecting processes fundamental to normal brain maturation such as myelination, synaptic pruning, and neuronal remodeling [46].

It has been assumed that multisystem biological dysregulations may take place in the peripheral blood of individuals with schizophrenia [47]. Consistent evidence suggests that circulating immune system proteins, including the acute phase response signaling pathway, are altered in first-episode psychosis (FEP) and people with a high risk of psychosis [48, 49]. Meta-analytical evidence shows elevated levels of IL-6 in the peripheral blood and cerebrospinal fluid (CSF) of individuals with FEP and diagnosis of schizophrenia [50–52]. A recent Mendelian randomization study found that genetically determined IL-6 was associated with changes in brain structure, with stronger associations in the middle temporal gyrus than in the whole brain, potentially involved in neurodevelopmental disorders, including schizophrenia and autism [53]. Therefore, an association between psychosis and a genetic variant that regulates IL-6 activity suggests that the IL-6/IL-6R pathway may be causally related to schizophrenia [54]. Furthermore, circulating levels of IL-6 have also been shown to decrease after antipsychotic treatment, hypothesizing their use as possible biomarkers of response to treatment in schizophrenia [50]. Reduced variability in IL-6 levels was reported in drug-naïve FEP subjects, supporting the hypothesis that an immune alteration may represent the core component of the pathophysiology of the disease [55]. Individuals at clinical risk of psychosis may present subclinical inflammation primarily related to the presence of depressive symptoms [19, 56]. Since a reduction in IL-6 levels is associated with improvement in depressive symptoms, it has been proposed that antidepressants may affect circulating IL-6 levels in individuals with FEP [57]. Furthermore, IL-10 levels decreased in advanced-stage patients and a tendency to decrease in the initial stage was found, reinforcing the hypothesis of evaluating this parameter in individuals at very high risk of developing psychosis and in the FEP [58]. Recently, a meta-analysis study showed the relationship between inflammatory biomarkers and negative symptom severity in the antipsychotic naïve FEP population, suggesting that early anti-inflammatory pharmacological interventions may improve clinical outcome in FEP [59]. The inflammation hypothesis of schizophrenia molecular pathophysiology has been supported by the findings of intrathecal immunoglobulin synthesis and neuroimmune alterations at least in a small subgroup of psychotic patients, indicating chronic inflammation of CSF in psychotic disorders [60–62].

Activated microglial cells can increase the production and expression of pro-inflammatory cytokines, such as TNF-α and IL-1β, and neurotoxic substances, leading to neuroinflammatory and neurodegenerative processes. A preclinical model of schizophrenia demonstrated elevated expression of Toll-like receptor (TLR)-3 signaling, interferon (IFN)-α and IFN-β in the frontal cortex (FC) of adult offspring subjected to MIA by polyinosinic:polycytidylic acid (poly(I:C)), during fetal life with increased oxidative and nitrosative stress, and increased levels of TNF-α, IFN-α, and IFN-β in the FC [63]. For example, the activation of TLR-3 and pro-inflammatory cytokines such as IL-6 influence the development of schizophrenia-like behavior in adult offspring. Increased pro-inflammatory mediators can alter brain development and be associated with immune/inflammatory genes related to schizophrenia [63].

In summary, brain and systemic inflammation may significantly contribute to the development and progression of the disease, posing the basis for immune and inflammatory mediators to be considered as putative biomarkers of schizophrenia (Table 1) [43].

**Table 1:** Potential biomarkers linking inflammation to schizophrenia. BDNF, brain-derived neurotrophic factor; IL-1β, interleukin 1β; IL-6, interleukin 6; IL-23, interleukin 23; TBARS, thiobarbituric acid-reactive substances; PCC, protein carbonyl content; IL-10, interleukin 10; IFN-γ, interferon γ; IL-17, interleukin 17; IL-2, interleukin 2; TNF-α, tumor necrosis factor α; MRI, magnetic resonance imaging; FEP, first-episode psychosis; TRS, treatment-resistant schizophrenia; SCZ, schizophrenia; TGF-β, transforming growth factor-β

Authors	Study design	Subjects	Sample	Stage of disease	Biomarkers	Levels in SCZ
O’Connell et al., 2015 [64]	Cross-sectional	Nonpsychotic controls (*n* = 38) Individuals with SCZ (*n* = 35)	Serum	Patients treated with depot antipsychotic (range 0.2–10 years)	**IL-23**	**↑**
Allimuthu et al., 2021 [65]	Cross-sectional	Nonpsychotic controls (*n* = 40) Individuals with SCZ (*n* = 40)	Serum	Drug naïve and drug-free patients (mean duration of illness = 12 months)	**BDNF**	**↓**
					**IL-23**	**↑**
Pedrini et al., 2012 [58]	Meta-analysis	Patients at early stage (≤ 10 years) (*n* = 22) Patients at late stage (≥ 10 years) (*n* = 39) Their respective matched controls (*n* = 25) and (*n* = 32)	Serum	Early (≤ 10 years) and late stage of chronicity (≥ 10 years)	**TBARS, PCC, IL-6**	**↑**
					**IL-10**	**↓**
Pillinger et al., 2019 [55]	Cross- sectional	Nonpsychotic controls (*n* = 1470) Individuals with SCZ (*n* = 1263)	Blood	Antipsychotic-naïve patients with FEP	**IFN-γ** **IL-17** **TNF-α** **IL-6** **TGF-β**	**↑**
Dunleavy et al., 2022 [59]	Meta-analysis	FEP patients (*n* = 651) control subjects (*n* = 521)	Blood	antipsychotic naïve FEP (within first 5 years of duration of illness)	**IL-1β** **IL-6** **IL-2** **TNF- α**	**↑**
Green et al., 2011 [66]	Meta-analysis	Nonpsychotic controls (*n* = 970) Individuals with SCZ (*n* = 1114)	Serum	Drug naïve and medicated patients (duration of medication unknown)	**BDNF**	**↓**

### Cell-Mediated Response in Schizophrenia

Some evidence suggests that aberrations in cell-mediated immune pathways may contribute to the pathophysiology of schizophrenia. The main cellular components of the adaptive system include T and B lymphocytes. B lymphocytes produce antibodies, whereas T cells include components of both the Th1 and Th2 systems. The Th1 system is responsible for the production of pro-inflammatory cytokines such as IL-2, IFN-γ, and TNF-α. The Th2 system, on the other hand, promotes the generation and maintenance of antibody-mediated immune responses and generate anti-inflammatory cytokines such as IL-4, IL-10, and IL-13.

An infection or other lesions may induce immune-inflammatory response system (IRS) activation by inducing M1 macrophage and Th1 phenotypes, followed by activation of Th17 and Th2 and Regulatory T cells (Treg). Reduction of compensatory immunoregulatory reflex system (CIRS) may increase the vulnerability to develop IRS hyper-response after injury. Excessive release of M1, Th1, Th17, and Th2 cell products may exert neurotoxic effects related to cognitive impairment and symptoms of schizophrenia [67].

Transforming growth factor-β (TGF-β) coordinates the balance between the innate and adaptive immune systems. TGF-β inhibits T cell differentiation into Th1 or Th2. Treg, on the other hand, are essential components of immune tolerance, responsible for producing IL-10 and TGF-β, facilitating anti-inflammatory and immunosuppressive actions. It has been hypothesized that a hypofunctional Treg state generates systemic inflammation and a decreased response to the immunosuppressive effects of TGF-β. In the brain, TGF-β has been shown to play a critical role in multiple aspects of neurodevelopment, neurogenesis, and neuroprotection, including regulation of cell growth, differentiation, migration, synapse formation, and pruning. TGF-β is a potent inducer of astrocyte differentiation and is essential for the development, maintenance, and differentiation of microglia. During inflammatory conditions, microglial cells produce IL-10 that stimulates the synthesis and release of TGF-β from astrocytes, inducing microglial activation and subsequent inflammation. Th17 cells can also damage the blood–brain barrier, infiltrate the CNS, and contribute to neuroprogression by sustaining neuroinflammation [68]. Of interest, activation and maintenance of Th17 cells have been found to be pronounced in schizophrenia patients taking antipsychotic drugs [64, 69], possibly sustained by IL-23, a pro-inflammatory cytokine that belongs to the IL-12 cytokine family.

In sum, immune cells such as lymphocytes and monocytes may contribute in several ways to the proper development of the CNS, the neuronal modeling, and the dendritic spine formation. In this framework, schizophrenia has been conceptualized as a disease characterized by dysregulation of cell-mediated inflammatory processes, which eventually converge on defects of synaptic plasticity.

## Molecular Abnormalities Driven by Inflammatory Events Relevant for the Synapse

### Role of Overactive Microglia in Modulation of Synaptic Plasticity

Microglia include approximately 10–15% of all glial cells and are tissue-resident macrophages with crucial functions in the CNS, including neuronal support, cell removal, homeostasis, and regulation of synaptic plasticity [70]. Microglial overactivity has been associated with excessive synaptic loss and cognitive decline, while pathological reduction of microglial activity during neurodevelopment has been associated with reduced synaptic pruning and sustained synaptic connectivity deficits [71–73]. Microglial activation may represent a proximal mechanism by which both immunologic and neuroplasticity-related factors influence the pathophysiology of schizophrenia [22].Of interest, in the course of activation, microglia assume an ameboid morphology probably due to reduction in fractalkine signaling. Noteworthy, a genetic study demonstrated an association among Ala55Thr polymorphism in fractalkine gene and autism or schizophrenia phenotypes [74], pointing to the involvement of morphological changes of microglia in the pathophysiology of mental disorders (Table 2). One of the putative molecules implicated in the transformation of ramified into ameboid microglia is non-muscle myosin II, which is critical for cytoskeletal changes during migration and phagocytosis in inflammatory and demyelinating conditions [75]. Genetic mutations in these proteins and signaling factors regulating their activity have been recently connected with autism spectrum disorders, schizophrenia, and intellectual disability [76]. Therefore, genetic and environmental risk factors for schizophrenia converge to altered microglial function during development, adolescence, and adulthood, in response to systemic and central inflammation [77].

**Table 2:** Involvement of inflammation in molecular abnormalities relevant for the synapse organization and function. CX3CR1, C-X3-C Motif Chemokine Receptor 1; ZNF804-A, zinc-finger protein 804A; ANKRD1, Ankyrin Repeat Domain 1; PIK3AP1, Phosphoinositide-3-Kinase Adaptor Protein 1; INHBE, Inhibin Subunit Beta E; DDIT3, DNA Damage Inducible Transcript 3; TGF, transforming growth factor; iPSCs, induced pluripotent stem cells; SMAD4, SMAD Family Member 4; REST, RE1-silencing transcription factor; AMPAR, α-amino-3-hydroxy-5-methyl-4-isoxazolepropionic acid receptor; KYNA, kynurenic acid; QUIA, quinolinic acid; CSF, cerebrospinal fluid; NMDAR, N-methyl-D-aspartate receptor; IgG, immunoglobulins G; IL, interleukin; PANSS, Positive and Negative Syndrome Scale; TNF-α, tumor necrosis factor- α; N/A, not applicable

Topic	Authors	Study design	Outcome	Subjects	Clinical meaning
Glial cell modifications	Ishizuka et al., 2017 [74]	Genetic study in human	Association between CX3CR1-Ala55Thr mutation and schizophrenia phenotype	*N*=370	Inhibition of fractalkine-CX3CR1 signaling, resulting in an altered modulation of microglial activation
	Umeda-Yano et al. 2013 [85]	In vitro preclinical study	Expression of ZNF804-A gene and related genes founding that ZNF804-A-overexpression induces up-regulation of ANKRD1, PIK3AP1, INHBE and DDIT, genes linked to TGF-β signaling	N/A	Involvement of ZNF804-A gene in the susceptibility to schizophrenia via TGF-β signaling
	Liu et al., 2019 [86]	In vitro gain- and loss-of-function studies	Glial differentiation defect in schizophrenia patient-derived iPSCs	N/A	Involvement of SMAD4 and REST in the glial differentiation defect. Knock-down of these two genes exhibit a restoring normal glial differentiation
	Sellgren et al., 2019 [93]	In vitro preclinical study	Excessive synaptic pruning in schizophrenia	N/A	Involvement of risk-associated variants within the human complement component 4 locus in neuronal complement deposition and synapse uptake
Oxidative stress and synaptic plasticity	Corti et al., 2011 [100]	Post-mortem study in human	Expression in Brodmann Area 10 of proteins involved in glutamate neurotransmission: - reduction in GluR1 and GluR2 AMPAR subunits; - increase in Na^+^/K^+^ ATPase-α1	*N*=56	The fist result confirms the hypoglutamatergic tone; the second corroborates the view of an excessive glutamate release counteracting the reduced number/activity of ionotropic glutamatergic receptors
Immune response and neurotransmission	Hilmas et al., 2001 [121]	In vitro electrophysiological study	Interaction between KYNA and the nicotinic system in the brain	N/A	Hypoglutamatergic tones in schizophrenia could be responsible for elevated levels of KYNA relevant for the inhibition of α7 nAChR, suggesting a functionally cross-talk between the nicotinic cholinergic system and the kynurenine pathway in the brain
	Kegel et al., 2014 [123]	Clinical study	QUIN and KYNA levels in CSF CSF QUIN/KYNA ratio	*N*=48	CSF QUIN/KYNA ratio was lower in patients than in controls supporting an over­activated and imbalanced kynurenine pathway, favoring the production of KYNA over QUIN in patients with schizophrenia resulting in NMDAR hypofunction
	Gos et al., 2014 [124]	Post-mortem study in human	QUIN-immunoreactive microglial cells in the CA1, CA2/3, and dentate gyrus area of the posterior hippocampal formation	*N*=25	Impaired glutamatergic neurotransmission in the hippocampus of schizophrenia patients because of QUIN agonist action at NMDA receptor
	Muller et al., 1995 [125]	Clinical study	Albumin and IgG in the CSF of schizophrenia patients	*N*=27	Association between albumin and IgG levels in the CSF and the score at Scale for the Assessment of Negative Symptoms
	Glantz et al., 2000 [131]	Post-mortem study in human	Dendritic spine density on prefrontal cortical pyramidal neurons	*N*=45	Decrease in dendritic spine density of schizophrenia patients compared to healthy control (by 23%) and other psychiatric conditions (by 16%). These findings are coherent with hypothesis that the number of cortical and thalamic inputs are altered in schizophrenia patients
	Meisenzahl et al., 2001 [132]	Clinical magnetic resonance imaging study	Association between IL -1β polymorphism (T/T or T/C) and schizophrenia	*N*=92	Structural brain alterations, including deficits in gray and white matter in patients with schizophrenia carrying polymorphism C511T
	Ellman et al., 2010 [133]	Clinical study	Prenatal exposure to IL-8 during second/third trimesters of pregnancy and structural neuroanatomical alterations	*N*=23	The authors found structural alterations, previously associated to schizophrenia, including increase in ventricular CSF, decreases in left entorhinal cortex and right posterior cingulate volumes
	Potter et al., 1999 [134]	In vitro preclinical study	Effect of a combination of the cytokines IL-1, IL-11, leukemia inhibitory factor, and glial cell line-derived neurotrophic factor on conversion of mesencephalic-derived progenitor cells into dopamine neurons	N/A	Exposure to these cytokines is responsible for driving the conversion of progenitor cells into dopamine neurons
	Ling et al., 1998 [135]	In vitro preclinical study	Effect of several cytokines in the differentiation of rat dopamine neurons	N/A	Exposure to IL-1 results to be implicated in the differentiation of dopamine neurons
	Kabiersch et al., 1998 [136]	In vivo preclinical study	Effect of IL-1 administration in mice and dopamine content in the hypothalamus in adulthood	Not retrieved	Increased production of IL-1β during inflammatory processes in the perinatal period could be responsible for long-lasting, and probably permanent, alterations in neurotransmitter systems
	Jarskog et al., 1997 [137]	In vitro preclinical study	Effect of exposure at IL -1β, IL-6, and TNF-α in embryonic rat dopamine and serotonin neuronal	N/A	High doses of cytokine are responsible for alteration in cells survival
	Ji et al., 2022 [142]	Clinical magnetic resonance imaging study	Evaluation of peripheral complement and cortical thickness	*N*=165	Inverse association between peripheral complement and cortical thickness but neurobiological consequences are still to be clarify

Microglial activation was estimated by positron emission tomography (PET) with PK11195, a ligand also known as the mitochondrial 18 kDa translocator protein (TSPO) [78]. In schizophrenia, increased PK11195 binding potential was found to be a marker of inflammatory processes in the CNS [79]. As observed in a PET study by Takano et al., positive symptoms and disease duration in schizophrenia positively correlated with the cortical DAA1106 binding underlying microglial activation [80]. Microglia are activated by abnormal release of TGF-β, produced in astrocytes cells [81]. TGF-β negatively regulates microglia under physiological conditions [82, 83] but, when increased, results in microglia activation, myeline phagocytosis, and subsequent synaptic pruning [84–86]. Excessive microglial pruning may reflect a reduced capacity of Treg regulatory functions and increased TGF-β release in schizophrenia [87]. Therefore, microglia respond to excessive TGF-β exposure with the expression of genes associated with phagocytosis of synapses and myelin and downregulation of neurotrophic factors as well as increased expression of the C4 implicated in the hypothesis of abnormal synaptic pruning in schizophrenia [82, 88, 89].

Indeed, activated microglia eliminate weak or inactive synapses, a process that critically depends on long-term potentiation (LTP) and other aspects of synaptic plasticity [90]. This role potentially links microglial activation with evidence of inherited structural variations and altered expression of genes involving NMDA-dependent plasticity in schizophrenia [2]. Overactive microglia may contribute to the pathophysiology of schizophrenia by decreasing spine and synapse density on glutamatergic cortical pyramidal neurons below a threshold critical for the integrated functioning of networks relevant to schizophrenia [91–93]. Furthermore, altered glutamatergic synaptic activity could impact the regulation of interneurons function and the balance between excitation and inhibition in cortical circuits, potentially leading to disinhibition of dopamine release control [90, 94, 95]. Indeed, hyperactivation of microglia can lead to excessive synaptic pruning, loss of cortical grey matter, and dysfunction in prefrontal cortex (PFC) and hippocampus, responsible for cognitive and negative symptoms of schizophrenia [2]. On the other hand, dysregulation of cortical functions can result in disinhibition of the subcortical dopaminergic system, thus promoting the emergence of positive psychotic symptoms in schizophrenia [2]. Therefore, dysregulated microglia may contribute to the decline of gray matter and altered functional connectivity observed in schizophrenia, plausibly through pruning of weak synapses [96]. In this framework, targeting regulators of microglia activation would be indicated as a potential early therapeutic strategy in treating schizophrenia [97].

### Role of Oxidative Stress in Modulation of Synaptic Plasticity

**Fig. 3:**
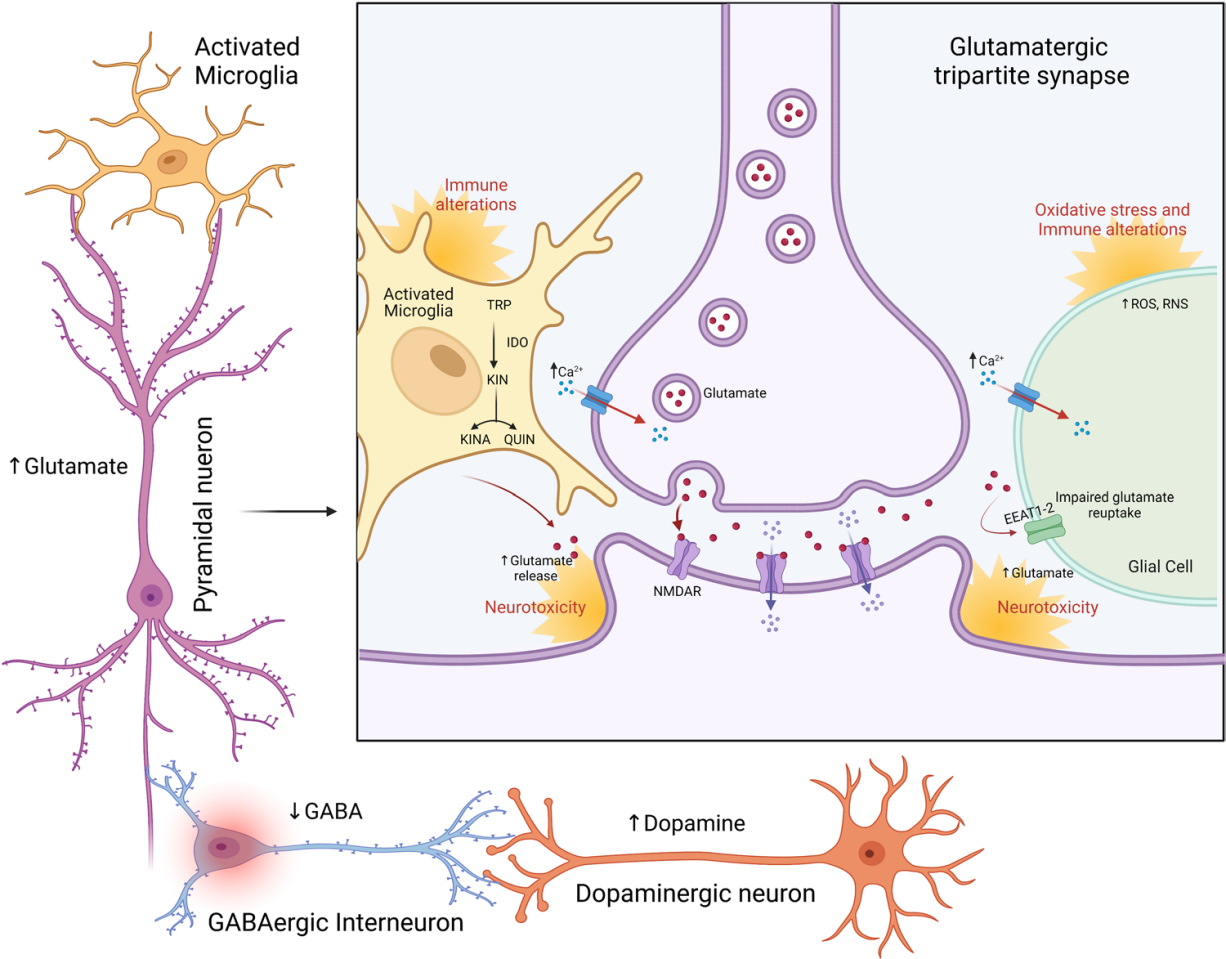
Overview of the oxidative stress and immune alterations influence on tripartite synapse in schizophrenia. Chronic oxidative stress may trigger multiple intracellular changes responsible for the increase in neuronal Ca2 + influx and therefore accumulation of ROS and RNS, disrupting synaptic transmission. The action of immune response on glial cells can cause an impairment of glutamate reuptake, inducing a further enhancing of neuronal Ca2 + influx, while in neurons can directly alter the membrane delivery of the AMPAR and NMDAR. KYNA and QUIN, the neuroactive metabolites of TRP/KYNA pathway, act as NMDAR antagonists and agonists, respectively. Following the glutamatergic hypothesis of schizophrenia, has been suggested the imbalance in KYNA pathway, promoting the production of KINA over QUIN resulting in microglial activation and KINA-mediated neurotoxicity. Moreover, GABAergic inhibitory interneurons dysfunction may induce a glutamate storm from excitatory glutamatergic cortical pyramidal neurons and a subcortical dopamine storm. GABA (γ-aminobutyric acid); TRP (tryptophan); IDO (indoleamine 2,3-dioxygenase); KIN (kynurenine); KINA (kynurenic acid); QUIN (quinolinic acid); ROS (reactive oxidative species); RNS (nitrosative species). Created with BioRender.com

Chronic oxidative stress may trigger multiple intracellular changes responsible for the increase in neuronal Ca^2+^ influx and therefore generate an intracellular accumulation of reactive oxidative species (ROS) and nitrosative species (RNS), disrupting synaptic transmission in the brain [98]. Moreover, these molecules alter Na^+^/K^+^-ATPase (NKA) activity in the FC of rats, an effect associated with an increased inflammatory response and oxidative and nitrosative damage. NKA is a membrane-bound protein that maintains transmembrane ion gradients that are crucial for the correct function of the CNS [98]. NKA is sensitive to membrane damage and evidence indicates the role of nitric oxide (NO) and free radicals in deteriorating its activity [99]. Disturbances in NKA function may result in severe psychiatric disorders such as schizophrenia [100] (Table 2). Of interest, the FC is a critical region of the brain for emotional regulation, decision-making, learning, and cognition [101]. A cluster of similar effects in this area has been observed in both chronically stressed animal models and humans, associated with dendritic shrinkage, impaired neuronal functioning, and elevated pro-inflammatory cytokine levels, as well as the increase in glucocorticoid signaling [102, 103]. Furthermore, several studies have shown that the oxidative stress and lipid peroxidation which has been reported in the hippocampus and FC of rats exposed to repeated stress is due to dysregulation of the hypothalamic–pituitary–adrenal (HPA) axis [104]. The dysfunction of the HPA axis and related activation of IRS could be associated with neurodevelopmental damage in brain areas involving glutamatergic neurotransmission [105]. HPA-axis dysregulation associated with hypercortisolemia has been reported in psychotic patients and might be elicited by pro-inflammatory cytokines such as IL-1, IL-6, and TNF-α in schizophrenia [105]. The action of TNF-α on glial cells can cause an impairment of glutamate reuptake, inducing an increase in neuronal Ca^2+^ influx, while in neurons can directly modulate the membrane delivery of the α-amino-3-hydroxy-5-methyl-4-isoxazolepropionic acid receptor (AMPAR) and N-methyl-D-aspartate receptor (NMDAR), further enhancing the neuronal Ca^2+^ influx [106]. In MIA offspring, the altered balance of Ca^2+^ levels in astroglial cells results in abnormalities in the structure and morphology of astrocytes, whereas the increase in glutamate levels is liable for excitotoxicity and reduced survival of neurons in the hippocampus [107] (Fig. 3).

This scenario promotes the activator protein 1 (AP-1) via MAPK cascade, which increases the transcription of inducible nitric oxide synthase (iNOS) and cyclooxygenase (COX)-2, generating ROS and RNS, as previously mentioned [108–110]. Chronic stress has been suggested to increase both neuroinflammatory parameters (TNF-α expression and AP-1 DNA binding) and iNOS and COX-2 activation in rats, converging on the impairment of NKA activity. Therefore, the alteration of NKA activity might be the molecular underpinning of the stress-related neuropsychiatric disorders, such as schizophrenia [111]. Serum indicators of oxidative stress and inflammatory cytokines may act as biological markers both in the early and late stages of chronic schizophrenia.

Specifically, thiobarbituric acid–reactive substances (TBARS) and protein carbonyl content (PCC) levels have been reported significantly higher in patients with schizophrenia than in controls, with no differences in total reactive antioxidant potential (TRAP) and TNF-α levels among patients at early and late stages. In summary, increased redox status together with impaired anti-oxidative stress defense may synergistically act as an accelerator of neuronal degeneration in schizophrenia [58].

### The Interaction Between Immune Response and Neurotransmission in Schizophrenia

It has been shown that dopamine has a role as a regulator of inflammation. Dopamine can regulate the activity, migration, differentiation, and proliferation of immune cells, including T cells, microglial cells, and peripheral monocytes, which contribute to cognitive functions [112–115] Therefore, alterations in dopaminergic transmission could influence the inflammatory response of immune cells and consequently the behavioral functions in schizophrenia [116]. Preclinical evidence shows that immune activation affects several neurodevelopmental processes, including dopaminergic and glutamatergic neurotransmission. The chronic subcutaneous administration of IFN-α in rhesus monkeys (*Macaca mulatta*) has been found associated with reduced dopamine release in the striatum, resulting in anhedonia and supporting the effect of inflammation on dopaminergic neurotransmission [117]. Immune alterations may also involve several neurotransmitter systems implicated in the pathophysiology of schizophrenia, acting on tryptophan (TRP)/kynurenine (KYN) metabolism. TRP metabolism may influence serotonergic and glutamatergic neurotransmission via activation or inhibition of the enzyme indoleamine 2,3-dioxygenase (IDO). The activated immune system can modulate IDO activity through the release of cytokines such as IFN-γ or TNF-α [118]. IDO is the rate-limiting enzyme converting TRP, the precursor of serotonin, into KYN. Therefore, IDO inhibition leads to an increase in serotonin availability. On the other hand, kynurenic acid (KYNA) and quinolinic acid (QUIN), the neuroactive metabolites of TRP/KYN pathway, act as NMDAR antagonist or agonist, respectively [119–124] (Table 2). KYNA acts as a blocker of the glycine binding site on the NMDAR, reproducing glutamatergic hypofunction. Conversely, QUIN is an NMDAR agonist exerting neurotoxic effects. According to the glutamatergic hypothesis of schizophrenia, some studies have pointed to an imbalance in KYNA pathway, promoting the production of KYNA over QUIN in the CSF and microglial cells of the hippocampus in schizophrenia [117, 119, 125–127] (Fig. 3). It has been hypothesized that accumulation of KYNA levels in the CNS affects dopaminergic activity [128]. The atypical antipsychotic clozapine, however, showed inhibitory effects on the activity of dopaminergic neurons in the midbrain [129], which are mediated by its action at the glycine binding site located on NMDAR [17], thus mitigating the NMDAR hypofunction induced by KYNA, improving the symptom dimension of schizophrenia [129].

Brain-derived neurotrophic factor (BDNF) is a neurotrophin that regulates neuronal survival and growth, which, in post-mortem studies, has been found to be reduced in the PFC of schizophrenia patients [66, 130]. Low BDNF reduces the proliferation of synapses or causes excessive pruning of synapses, leading to impaired synaptic plasticity and thus increasing the severity of the disease [131]. A clinical study found serum levels of BDNF negatively associated with IL-23 and disease severity, supporting the role of inflammation in modulating the growth, survival, and maintenance of synaptic connections [65]. The relationship between brain volume loss and an increased levels of the immune markers IL-1β and IL-8 has been described in the literature [132, 133]. IL-1β, which can induce the conversion of rat mesencephalic progenitor cells into a dopaminergic phenotype, and IL-6, which decrease the survival of fetal brain serotonergic neurons, both significantly influence the neurotransmitter systems involved in schizophrenia. Moreover, the administration of IL-1β after birth affects dopaminergic neurotransmission in adulthood [134–138]. Regarding glutamate neurotransmission, the action of IL-1β has the potential to act on both excitatory and inhibitory components, modulating intracellular signaling in the brain, and altering the expression of genes encoding enzymes that regulate glutamate neurotransmission. Since schizophrenia is related to a dysfunction of dopaminergic and glutamatergic brain circuits, it has been argued that IL-1β may causally link inflammatory processes to dopaminergic/glutamatergic dysfunctions [43].

The role of tetrahydrobiopterin (BH4), an essential enzyme cofactor for the synthesis of tyrosine and dopamine, regulated by inflammatory cytokines, could explain the interaction between the dopaminergic and immune systems. Indeed, cytokines can regulate the expression of GTP-cyclohydrolase 1 (GCH-1), an enzyme required for BH4 production, promoting dopamine biosynthesis [139]. During inflammation, ROS and iNOS activity leads to a reduction in BH4 with a subsequent decrease in dopamine synthesis. Similarly, IFN-α, IL-6, and cardiotrophin-1 may reduce BH4 levels, while IL-1β, IFN-γ, and TNF-α may increase BH4 availability, thus playing opposing effects on dopaminergic system [140, 141].

It has been found that at embryonic stages, TGF-β signaling induces the release of extracellular matrix remodeling enzymes, such as metalloproteinase 9 (MMP9). MMP9 reduces the inhibitory activity of GABAergic interneurons by disrupting the related perineuronal networks (PNNs), which enable their rapid spiking, thus contributing to GABAergic dysfunctions already reported in schizophrenia [87]. GABAergic inhibitory interneurons dysfunction could induce a significant release of glutamate (glutamate storm) from excitatory glutamatergic cortical pyramidal neurons and a subcortical hyperdopaminergic state (dopamine storm). In particular, some authors have proposed that the increase in extracellular glutamate may act as a brain trigger in neuroprogression [5] (Fig. 3).

A potential link between inflammation, imbalanced glutamate-dopamine interaction, and post-synaptic changes is supported by recent results showing a significant increase in mRNA encoding complement receptors (C5ar1, CR1, CR3a), regulators (CD55, C59), and proteins (C3, C3b, C4) in the plasma of schizophrenia patients compared to healthy controls. The increased mRNA expression of inflammatory mediators was associated with reduced cortical thickness in schizophrenia patients. Therefore, the complement cascade upregulation significantly affected cerebral cortex integrity and functional connectivity, relevant for cognitive impairment schizophrenia [142].

## Animal Inflammatory Models of Schizophrenia

The relevance of neuroinflammation for schizophrenia pathogenesis led to peculiar animal modeling based on the induction of early immune responses to reproduce schizophrenia-like behavioral and molecular phenotypes in rodents. Among these well-recognized animal models of schizophrenia, the maternal immune challenge and neonatal immune activation should be mentioned. Maternal infection gained interest in light of epidemiological evidence linking the increased risk of schizophrenia with birth in the winter and spring months, probably due to seasonal variation in influenza epidemics [32].

MIA can be triggered by the injection of the viral mimic poly(I:C), a synthetic double-stranded RNA, or bacterial mimic lipopolysaccharide (LPS), which replicates the external leaflet of Gram-negative bacteria outer membrane. Administration of these immunostimulant compounds during pregnancy induces massive immune responses, the release of pro-inflammatory cytokines, and subsequent dysregulation of the maternal immune milieu, thus affecting fetal neurodevelopment. MIA protocols foresee single or multiple poly(I:C) intraperitoneal injection with different dosages (5–20 mg/kg) at several stages of pregnancy in mice or rats ranging from the gestational day (GD) 8.5 to GD 18.5, approximately corresponding to the first-to-second and the second-to-third trimesters of human pregnancy, respectively [143]. Poly(I:C) interacts with the TLR-3 expressed on the surface of host B cells, macrophages, and dendritic cells, but it can also be recognized directly by cytosolic sensors. However, the engagement of the downstream pathways leads to an imbalance between pro-and anti-inflammatory cytokines in the utero environment, relevant for embryonic neurodevelopment. Similarly, intramuscular injection of turpentine oil into pregnant dams produces a model of maternal aseptic inflammation, with localized necrotic damage and subsequent induction of TNF-α and IL-1β at the site of injury, as well as the release of circulating IL-6 [144, 145]. Although this cytokine is crucial for normal embryo implantation, IL-6 appears to be the main circulating mediator of the inflammatory response in MIA models, and its upregulation leads to early damage of dopaminergic neurons and neurodevelopmental disorders in the offspring [146]. Furthermore, maternal injection of IL-6 alone is sufficient to induce the behavioral abnormalities seen in other inflammatory models. On the other hand, when IL-6 is eliminated by co-administration of cytokine neutralizing antibodies targeted against IL-6, or by genetic manipulation, schizophrenia-like abnormalities are not detectable in the offspring [63]. MIA produces offspring exhibiting various abnormalities in behavior, sensorimotor gating, gene expression, and microglial activity (Table 3). In particular, MIA results in behavioral impairments recapitulating schizophrenia as well as autism spectrum disorder abnormalities, such as reduced sociability, altered ultrasonic vocalization, enhanced repetitive and anxiety-like behavior, and learning and memory deficits [147, 148]. Social behavior impairments included reduced ultrasonic vocalizations of pups, as well as changes in the vocalization patterns with increased short and complex syllables, as well as low levels of harmonic syllables. Moreover, a deficit in scent-marking has been detected in adult mice in response to female urine, probably underlying a lack of sexual interest in MIA offspring [149]. Anxiety-like and repetitive/compulsive behaviors have been detected in the marble burying test, as reflected by burying novel objects placed in the cage and repetitive digging behavior, as well as excessive grooming or head bobbing [149–152]. MIA offspring exhibit also reduced exploration in open field, reduced performance in novel object recognition task, and reduced cognitive flexibility [153–155]. Additionally, sensorimotor deficits have been repeatedly reported in MIA offspring, as reflected by impairments in pre-pulse inhibition (PPI), paralleling those observed in schizophrenia patients. In fact, PPI, namely a decline in the startle reflex, is a common measure of the ability to temporarily adapt to intense sensory stimuli when a preceding weaker pre-stimulus (pre-pulse) is given. Patients suffering from schizophrenia generally exhibit a PPI deficit, due to oversensitivity to external stimuli and failure to perform pre-attentive filtering functions aimed at discriminating relevant and irrelevant sensory information [156, 157]. PPI deficits are therefore assumed to be a hallmark feature of animal models of schizophrenia and are evident in both adult and juvenile MIA animals [158]. Furthermore, to support the relevance of MIA models to the pathophysiology of schizophrenia, it has been observed that PPI deficits are reversed by acute or chronic antipsychotic treatment with haloperidol, olanzapine, risperidone, and clozapine in adolescent or adult rodents [159, 160]. Similarly, another measure of information gating, also known as “latent inhibition,” appears to be disrupted in the adult offspring of dams challenged with poly(I:C). Latent inhibition is the phenomenon whereby pre-exposing to a non-reinforced stimulus retards subsequent response to the same stimulus when it is later paired with a salient event [161]. Therefore, the abolished latent inhibition in MIA offspring reflects the inability to ignore irrelevant stimuli and the cognitive deficits characteristic of schizophrenia [162, 163]. Noteworthy, the effects of MIA are also detectable at a morphological level based on microglia aspect, density, and distribution, as well as clustering, arborization, and transformation into a reactive state [164, 165]. It should be noted that microglial dystrophy has been proposed to contribute to the pathogenesis of schizophrenia and progress during the disease, resulting in a subsequent increase in oxidative stress and mitochondrial dysfunctions [5, 166]. Moreover, microglia activation following maternal poly(I:C) challenges may be more pronounced in male than female offspring, mirroring the higher incidence of schizophrenia in males as well as more severe clinical features [167, 168]. Proteomic analysis of poly(I:C)-treated rodents showed significant changes in the protein expression of 10-formyltetrahydrofolate dehydrogenase (ALDH1L1) and collapsin response mediator protein 5 (CRMP5) [160]. More specifically, ALDH1L1 is an enzyme converting 10-formyl-tetrahydrofolate to tetrahydrofolate and carbon dioxide in a NADP^+^-dependent reaction, which is expressed in glial cells and astrocytes and has been found to increase in schizophrenia patients [169]. On the other hand, CRMP5 is the protein responsible for axonal guidance control, linking the cytoskeleton to the growth cone during neuronal development [170]. Therefore, these findings suggest that MIA offspring exhibit crucial abnormalities in protein expression, potentially underlying dysfunctions in neuron-astrocyte interactions. MIA models are associated with a wide range of histopathological and neurochemical abnormalities in regions involved in the pathophysiology of schizophrenia. Poly(I:C) offspring exhibit moderate-severe cell loss and pyknotic-like neuronal profiles mainly affecting the hippocampal cornu ammonis (CA)-1 and CA-3 regions, the dentate gyrus, and the entorhinal cortex. The damage to limbic structures results in post-pubertal striatal dopamine hyperactivity, namely, amphetamine-induced or spontaneous excess of dopamine release, probably due to disrupted temporo-limbic-striatal interplay [162]. It should be noted that the tardive emergence of dopaminergic hyperfunction, not detectable before puberty, may reflect the neurodevelopmental nature of the disease, according to Weinberger’s hypothesis [171]. An increase in dopamine content has been detected in the PFC, nucleus accumbens, and globus pallidus in the MIA newborn offspring of dams injected with GD-9 poly(I:C), while glutamate and GABA content remained unchanged [172]. The increase in dopamine content may be due to the increased activity of tyrosine hydroxylase (TH), the enzyme which catalyzes the rate limiting step in dopamine synthesis. In fact, increased number of TH immunoreactive cells in the ventral tegmental area and TH-positive terminals in the striatum have been noticed in MIA offspring [173]. Furthermore, high-performance liquid chromatography analysis revealed an increase in the striatal concentration of dopamine metabolites, including 3,4-dihydroxyphenylacetic acid and homovanillic acid in poly(I:C) mice, indicating an increase in dopamine turnover. In the same study, Ozawa et al. also demonstrated decreased binding of striatal dopamine D2 receptors (D2Rs) from adult poly(I:C) mice compared to controls [174]. These abnormalities may represent a physiological adaptation to the increased subcortical dopamine activity elicited by the prenatal immune challenge. Mundorf et al. found a reduction in D2R and dopamine D1 receptor (D1R) expression in the PFC of adult MIA, while other authors found a post-pubertal increase in D2R expression in the nucleus accumbens [175, 176]. These findings may indicate that the increase in dopamine activity elicited by prenatal immune challenge may affect the mesolimbic but not the mesocortical pathway. Intriguingly, the dopaminergic dysfunctions observed in the direct descendants of poly(I:C)-treated mothers are opposed to those identified in the subsequent generations. While the first-generation offspring showed a hyperdopaminergic state, the second and third generation offspring of immune-challenged ancestors showed indirect signs of decreased dopaminergic function, such as reduced sensitivity to amphetamine and decreased TH expression [177]. However, albeit of opposite sign, dopaminergic alterations can be transmitted across generations. Furthermore, also glutamatergic neurotransmission was found to be impaired in MIA rodents. MIA offspring exhibited impaired glutamate release evoked by depolarization in the hippocampus and reduced expression of NMDAR in the PFC and hippocampus [178]. Young adult offspring showed an increase in all subunits of the NMDAR, as well as post-synaptic density protein 95 (PSD-95) levels in the PFC, while only the NMDAR subunit 2A (NR2A) increased in the hippocampus [179, 180]. It should be noted that PSD-95 is a scaffolding protein of post-synaptic density (PSD), which is crucial for NMDAR downstream signaling and synaptic efficacy. Therefore, both the dopaminergic and glutamatergic systems exhibit several abnormalities in the MIA offspring, resulting in aberrant dopamine-glutamate interplay. MIA offspring showed also disturbances in the inhibitory neurotransmission, in particular a reduction in parvalbumin positive (PV +) cells, which in turn do not inhibit pyramidal excitatory neurons [181]. Although dopaminergic and glutamatergic abnormalities observed in MIA are reminiscent of those observed in schizophrenia pathophysiology, changes in GABAergic inhibitory signaling have been associated with both schizophrenia and autism [182]. The expression of several amino acid transporters, whose function is crucial in regulating fetal development, has been found to be altered in the placenta and fetal brain of poly(I:C) rodents. Of interest, alanine serine cysteine transporter 1 (ASCT1) and excitatory amino acid transporter (EAAT)-2 protein levels decreased in the placenta, as well as sodium-coupled neutral amino acid transporter 5 (SNAT5), excitatory amino acid transporter (EAAT)-1, and glycine transporter 1 **(**GLYT1) expression in the fetal brain, leading to subsequent widespread changes in free amino acid content [183]. As well known, placental EAAT-2 is responsible for glutamate transport between the fetal and maternal blood circulation, regulating glutamate excitotoxicity, which can be detrimental for the neurodevelopment. Similarly, GLYT1 is essential for glycine reuptake, thus terminating glycine-mediated inhibitory signaling [184]. Given their relevance to inhibitory/excitatory balance in early life, disruptions in the expression of amino acid transporters may be critical for the regulation of synaptic activity and the development of the CNS. MIA rats also exhibited altered cortical levels of presynaptic proteins, including synaptobrevin and syntaxin-1, which are key components of the N-ethylmaleimide-sensitive factor attachment protein receptor (SNARE) complex, responsible for the formation and release of vesicles. Abnormalities in the expression of pre and post-synaptic proteins were associated with ultrastructural changes in the synapses of rats exposed to LPS-induced MIA, such as reduced packing density of synaptic vesicles, swelling of the neuropils, blurred and thickened structures of the synaptic cleft, and abnormal synaptic membranes, as well as swollen endoplasmatic reticulum and altered architecture of mitochondrial cristae [180].

**Table 3:** Behavioral and molecular features recapitulating schizophrenia-like phenotypes observed in the offspring following maternal immune activation protocols. MIA, maternal immune activation; GD, gestational day; E, embryonic day; PolyI:C, polyinosinic:polycytidylic acid; LPS, lipopolysaccharide; i.p., intraperitoneal; i.v., intravenous; s.c., subcutaneous; Th, tyrosine hydroxylase; EGF, epidermal growth factor; GABA, γ-aminobutyric acid; NMDAR, N-methyl-D-aspartate receptor; PPI, pre-pulse inhibition; LI, latent inhibition; SN, substantia nigra; VTA, ventral tegmental area; PFC, prefrontal cortex; IL, interleukin; EAAT, excitatory amino acid transporter; SNAT, sodium-coupled neutral aminoacid transporter; GLYT, glycine transporter; ALDH1L1, aldehyde dehydrogenase 1 family member L1; CRMP5, collapsin response mediator protein 5; SERPINA3, serpin family A member 3; TNF-α, tumor necrosis factor α; SNARE, soluble N-ethylmaleimide-sensitive fusion protein attachment protein receptors; PSD-95, Postsynaptic density protein 95 kDa

Authors	MIA model	Outcome
Shi et al., 2003 [155]	Intranasal infusion with human influenza virus on GD9.5	In dams: mild lung consolidation In the adult offspring: altered exploratory behavior in both open-field and novel-object tests, reduced social interaction and impaired PPI
Baines et al. 2020 [143]	i.p. injection with poly(I:C) on GD8.5	In dams: decreased expression of the maternally imprinted genes *Mest*, *Sfrp2*, and *Dlk1* Latent placental development and reduced fetal growth
Malkova et al., 2012 [149]	i.p. injections with poly(I:C) on E10.5, 12.5 and 14.5	Decreased sociability, lower rate of ultrasonic vocalizations in response to social encounters, reduced scent marking in adult offspring
Nakamura et al., 2022 [154]	i.p. injection with poly(I:C) on GD9, 10, and 11 or GD 13, 14, and 15	In dams: increase in maternal serum IL-6 levels, which was higher in mice exposed to poly(I:C) during the early window In the offspring: female-specific disruptions to working memory and reduced perseverative behavior in mice exposed in the early window; male-specific alteration in working memory, and cognitive flexibility in mice exposed in the late window; increased fetal neuregulin/EGF pathway expression but reduced adult expression; reduction in *Grin2d* expression and discrete changes in the expression of GABAergic and dopaminergic pathway genes
Wolff et al., 2010 [158]	i.v. injection with poly(I:C) on GD15	Unaffected litter size; disruption of PPI in both juvenile and adult MIA rats
Zuckerman et al., 2003 [162]		Unaffected LI in the juvenile offspring, but disrupted in adulthood
Winter et al., 2009 [172]	i.v. injection with poly(I:C) on GD9	In the offspring: increase in dopamine levels and their metabolites in the PFC and lateral globus pallidus; decreased serotonin and its metabolite in the hippocampus, nucleus accumbens, and lateral globus pallidus
Meyer et al., 2008 [173]		Increase in the number of fetal mesolimbic dopaminergic neurons; changes in fetal expression of several genes known to be involved in dopamine neuron development, including *Shh*, *Fgf8*, as well as transcription factors Nurr1 and Pitx3
Vuillermot et al., 2010 [176]		Dopaminergic maldevelopment starting in the fetal stages of life, followed by postnatal dopaminergic abnormalities; increase in Th-positive dopamine cells in the SN of fetal and SN and VTA of adult offspring; increase in Nurr1-positive cells in the SN of fetal and adult offspring relative to control offspring
Hao et al., 2019 [179]		In dams: increase in blood levels of IL-6, IL-1β, and TNF-α In the offspring: increased locomotor activity in adolescence; increase in anxiety-related behavior in adulthood; PPI deficits, and progressive impairment in spatial exploration, spatial recognition memory, and working memory from adolescence to adulthood; age-related alteration of NMDA receptors in the prefrontal cortex and hippocampus from weaning to adulthood
Giovanoli et al., 2016 [186]		Postsynaptic hippocampal deficits in pubescence; PPI deficit, altered hippocampal IL-1β and synaptophysin levels in adult offspring
Hui et al., 2020 [165]	i.p. injection with poly(I:C) on E9.5	Sex-specific alterations in microglial pruning, complement system, neuronal health, inhibitory and excitatory synapses density and activity in the dentate gyrus of adult offspring, resulting in abnormal synaptic connectivity
Canetta et al., 2016 [181]	i.v. injection with poly(I:C) on E9	Reduced GABAergic transmission in the medial PFC of adult offspring, due to a selective decrease in functional connectivity between the PV class of interneurons and pyramidal cells
Mundorf et al., 2021 [175]	i.p. injection with poly(I:C) at GD15	Reduced DRD2 mRNA in PFC of adolescent, but not adult animals
Oh-Nishi et al., 2010 [187]	i.p. repeated injection with on GD15-17	Decrease pre-synaptic protein expression and altered electrophysiological synaptic functions in juvenile offspring
McColl et al., 2019 [183]	i.p. injection with poly(I:C) on GD14	Increased mRNA expression of several amino acid transporters in the placenta and fetal brain; decrease in protein levels of ASCT1 and EAAT2 in placenta; decrease of protein levels of SNAT5, EAAT1, and GLYT1 in fetal brain
Kitagawa et al., 2019 [160]	repeated s.c. injection with poly(I:C) on post-natal days 2–6	PPI deficit, emotional and cognitive dysfunction in the offspring; changes in the protein expression of ALDH1L1 and CRMP5 (astrocyte-neuron interaction molecules) in the hippocampus
Ozawa et al., 2005 [174]	repeated i.p. injection with poly(I:C) from E12 to E17	Increased subcortical dopamine function and cognitive impairment in the offspring
Weber-Stadlbauer et al., 2021 [177]	Injection of poly(I:C) on GD9	In first-generation offspring: signs of hyperdopaminergia, increased sensitivity to amphetamine, and increased expression of Th in the adult ventral midbrain; increased DNA methylation at the promoter region of Nurr1, in the sperm of first-generation MIA offspring In second-generation offspring: increased methylation at the promoter region of Nurr1 the ventral midbrain and reduced levels of Nurr1 protein In second- and third-generation offspring: blunted locomotor responses to amphetamine and reduced expression of Th
Ozaki et al., 2020 [164]	i.p. injection with poly(I:C) on E12 or E15	In dams: injections at both gestational days significantly increased the expression of IL-6 in both the maternal liver and placenta In the offspring: altered open field behavior in E12 PolyI:C-injected mice; reduced social behavior in both E12 and E15 PolyI:C; subtle differences in the level of postnatal microglial differentiation; increased the velocity of microglial tip movements in mice injected at both time points
Purves-Tyson et al., 2021 [188]	i.v. injection with poly(I:C) on GD17	Increased transcripts of immune markers (i.e., SERPINA 3, TNF-α, and IL1β) in the midbrain of the offspring
Aguilar-Valles et al., 2007 [144]	i.m. injection with turpentine on GD15-18	In dams: fever lasting for over 24 h. A significant rise in circulating IL-6 and prostaglandin E_2_ levels
Aguilar-Valles and Luheshi, 2011 [145]		In the adult offspring: a decrease in PPI of acoustic startle, increased latency in the cued task of the Morris-water maze, prolonged conditioned fear response and enhanced locomotor effect of amphetamine Increased Th expression in the nucleus accumbens of the adult offspring of mothers treated on GD15
Smith et al., 2007 [63]	i.p. injection with IL-6 on GD12.5	In the adult offspring: PPI and latent inhibition LI deficits
Fernández de Cossío et al., 2017 [151]	i.p. injection with LPS on E15	In pups: reduced vocalizations; increased spine density in granule cells and reduced expression of pruning molecules in males In the adult offspring: reduced social interest, increased number of stereo typies
Cieślik et al., 2020 [180]	i.p. injection with LPS on GD9.5	In dams: sickness behavior In the offspring: altered social behavior; increased inflammatory cytokines in blood and brain; microglia activation in brain cortex of adolescent rats; a wide set of ultrastructural abnormalities including neuropil swelling, reduced packing density of synaptic vesicles in the presynaptic area, blurred structure of synaptic cleft, changes in mitochondria and myelin structures in the somatosensory cortex; altered SNARE complex components and decrease in PSD-95 and scaffolding synaptic proteins in the brain cortex

**Fig. 4:**
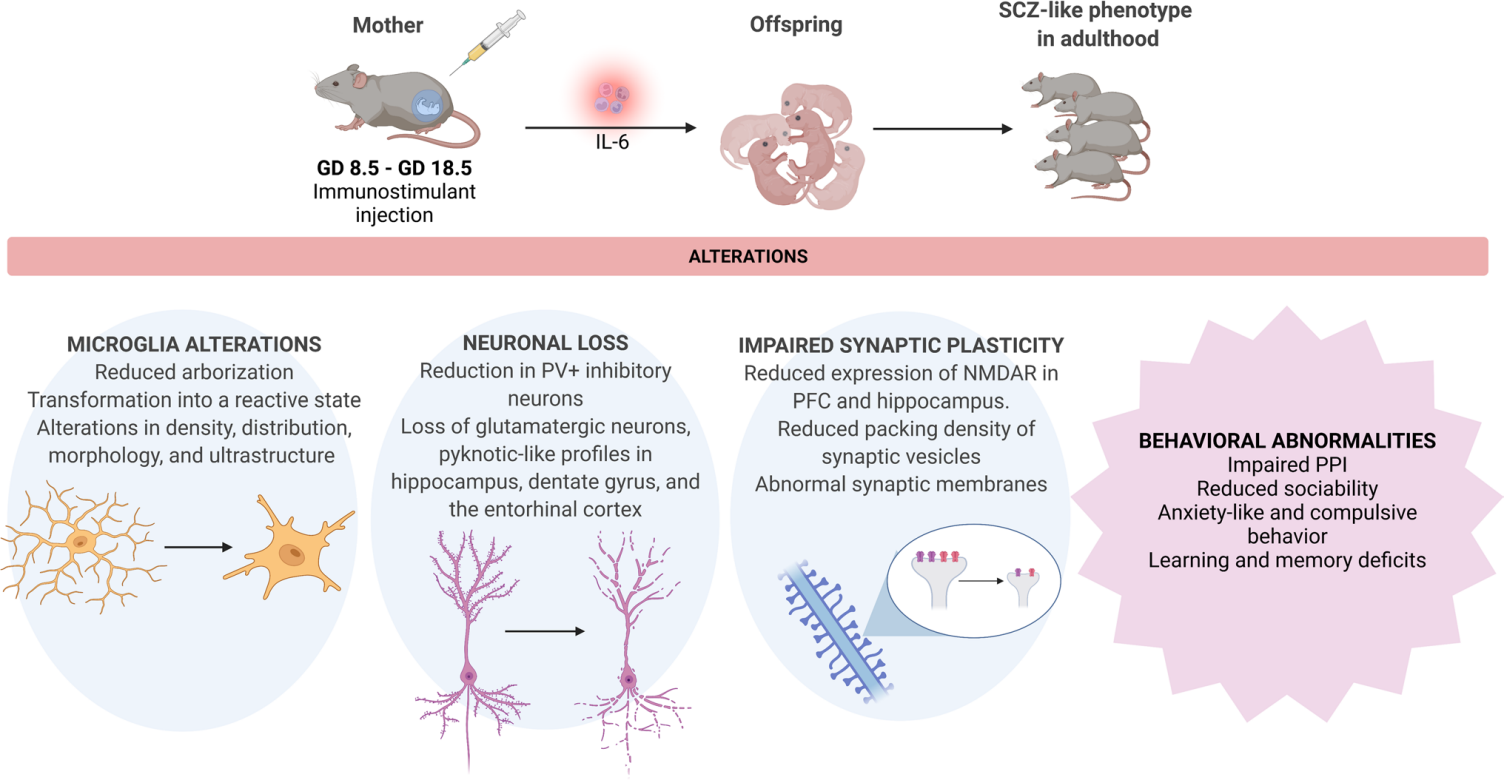
MIA rodent models are based on the observation that immune challenges experienced by the mother during gestation can exert inflammatory responses which disrupt fetal neurodevelopmental processes. In particular, MIA results in a wide range of neurochemical, histopathological, and behavioral alterations in the offspring that recapitulate the pathophysiology of schizophrenia. GD 8.5 (gestational day 8.5); GD 18.5 (gestational day 18.5); IL-6 (interleukin 6); SCZ (schizophrenia); PV + (parvalbumin-positive); NMDAR (N-methyl-D-aspartate receptor); PFC (prefrontal cortex); PPI (pre-pulse inhibition). Created with BioRender.com

In summary, MIA offspring closely recapitulate behavioral, electrophysiological, molecular, and genetic aspects of schizophrenia allowing to model at preclinical level a schizophrenia-like condition based on immune and inflammatory background, as well as to test pharmacological interventions that could putatively counterbalance brain abnormalities correlated to the model (Fig. 4). The major limitation of MIA is that early immune challenge in the absence of a specific pathogen may increase the risk of a broad spectrum of CNS changes that may be not exclusive of schizophrenia only [185].

## Influence of Inflammation on Synaptic Structures and Dendritic Spine Architecture in Schizophrenia

The neurobiology of schizophrenia has been associated with alterations in the PSD structure, which contributes to the abnormalities observed in the architecture of dendritic spines [189]. The PSD, a specialized ultrastructure that electron microscopy recognizes as a thickness of glutamatergic synapses, is crucial for the neurodevelopment. It is constituted by three orders of layered molecules, including metabotropic and ionotropic receptors, scaffolding proteins (i.e., PSD-95, Shank, Homer), cytoskeleton proteins (i.e., tubulin, actin, α-internexin), and enzymes that propagate and transduce the signaling from the cell surface to the downstream effectors in the intracellular compartment, resulting in synaptic maturation, signaling integration, and dendritic spine organization [190].

Therefore, early abnormalities of PSD composition are associated with a reduction in the number of dendritic spines and/or their rapid elimination, potentially leading to the development of neuropsychiatric disorders [191–194]. Dendritic spines mediate most of the excitatory neurotransmission in the CNS and have a critical function in the brain. Spines are biochemical compartments that may electrically regulate synaptic potentials through isolated depolarization in individual spines, reflecting localized synaptic activation. A significant voltage gradient between the dendritic spine and the dendritic shaft indicates that spines may constitute elementary electrical compartments [195] whose regulation could be crucial for synaptic function [196], synaptic plasticity [197], dendritic integration [198], and then could be altered in mental and neurological diseases [199]. Coiro et al. have shown that prenatal inflammatory challenges may account for persistent alterations in adulthood, such as reduction in dendritic spine density, dynamics, and excitatory and inhibitory synaptic functions, resulting in altered processing of incoming inputs [200]. Beyond PSD involvement in the pathophysiology of mental diseases and, in particular, schizophrenia, the presynaptic structure also plays a critical role. Presynaptic structures, together with PSD and astrocytic processes, constitute the functional unit known as “tripartite synapse,” which is relevant to modulate neuroplasticity events [201]. In this context, astrocytic leaflets, filling the space between the presynaptic bouton and the dendritic spine, provide a functional interface capable of organizing active synaptic connections. All these structures composing the tripartite synapse are extremely sensitive to environmental inflammatory manipulations which may lead to circuitry alterations underlying schizophrenia. In this section, we evaluate the current literature on the role of inflammation in modulating and restructuring synaptic architectures, with particular attention to post-synaptic alterations at both the morphological and molecular levels.

### Inflammation and Structural Changes in PSD Composition

The structural regulation of the dendritic spine, namely the protein network acting as a scaffold for stably positioning channels, cell adhesion proteins, and other PSD proteins, is heavily influenced by stimuli such as inflammation and immune responses (Table 4).

**Table 4:** Dendritic spine alteration linked to inflammation. PSD-95, Postsynaptic density protein 95 kDa; PFC, prefrontal cortex; IL, interleukin; F-actin, Filamentous actin; LTP, long-term potentiation; SynGap, synaptic Ras GTPase-activating protein; LPS, lipopolysaccharide; β2m, β2 microglobulin; MHCI, major histocompatibility complex I; sMHCI, surface MHCI; Poly(I:C), polyinosinic:polycytidylic acid; GABA, gamma-aminobutyric acid; 5mC, 5-

Authors	Study design	Brain region	Animal model	Molecular/structural modifications at dendritic spine	Functional implications
Tong et al., 2012 [204]	In vitro preclinical study	HC	Exposure of cultured rat hippocampal slices to IL-1β	IL-1β suppresses the phosphorylation of cofilin, which is required for actin polymerization in spines; IL-1β also prevented the formation of F-actin in spines and impaired the consolidation of LTP	IL-1β may prevent formation of F-actin in dendritic spines, impairing LTP consolidation and impair synaptic plasticity
Kolmogorova et al., 2021 [208]	In vivo preclinical study	HC, PFC, hypothalamus, and cerebellum	Systemic LPS administration in pubertal rats	PSD-95 levels were altered one-week post-treatment by the pubertal LPS treatment; males showed increased PSD-95 expression in the hippocampus	LPS induces changes in expression of synaptic plasticity proteins, with sex-specific vulnerability
Cui et al., 2015 [207]	In vivo preclinical study	N/A	LPS injection in male Sprague–Dawley rats	Increase in the expression of Homer1b/c in rat brain	LPS can induce changes in the expression of Homer1b/c
Giovanoli et al., 2016 [186]	In vivo preclinical study	HC	Maternal immune activation in mice	Altered synaptophysin and reduction in PSD-95 levels in adult age Decreased SynGap density in the dorsal CA region at pubescent but not adult age	Prenatal immune activation induces an adult onset of presynaptic hippocampal deficits
Cieślik et al., 2020 [180]	In vivo preclinical study	Somatosensory cortex	Maternal immune activation in mice	Synaptic ultrastructural abnormalities, altered SNARE complex components and decrease in PSD-95 and scaffolding synaptic proteins	Prenatal immune activation causes age-dependent hippocampal post-synaptic deficits in the offspring
Pierre et al., 2022 [217]	In vitro preclinical study	N/A	Primary neonatal cell co-cultures of astrocytes and microglia were treated with LPS	LPS-induced inflammation led to abundant IL-1 expression, IL-1 inhibition had no significant impact on in vitro modulation of microglia and astrocyte activation pathways	LPS induced an astrocytic markers profile during the early phase and continuous LPS exposure of inflammation; synaptic loss
Glynn et al., 2011 [218]	In vitro and in vivo preclinical studies	Cortical neurons	Cultured neurons of β2m knockdown, β2m^−/ −^ mice	MHCI negatively regulates the density and function of cortical synapses during their initial establishment*:* in vitro*,* decreasing sMHCI on neurons increases glutamatergic and GABAergic synapse density; i*n vivo*, synapse density is higher throughout development in β2m − / − mice	Acute changes in sMHCI and activity alter synapse density exclusively in early postnatal development; MHCI molecules modulate activity-dependent refinement and plasticity
Labouesse et al., 2015 [219]	In vivo preclinical study	mPFC	Poly(I:C) offspring	Prenatal immune activation increased prefrontal levels of 5mC and 5hmC in the promoter region of GAD1, which encodes the GAD67; the early-life challenge increased 5mC levels at the promoter region of GAD2, which encodes the GAD65; the elevation of GAD1 and GAD2 promoter binding of MeCP2 reduced GAD67 and GAD65 mRNA expression	Epigenetic modifications represent a mechanism whereby maternal infection induces GABAergic impairments in the offspring; hypermethylation of GAD1 and GAD2 promoters may linking prenatal infection to presynaptic GABAergic impairments and correlated with prenatal infection-induced impairments in working memory and social interaction
Lee et al., 2014 [220]	In vivo preclinical study	RGCs and LGN neurons	Mice lacking both H2-Kb and H2-Db (KbDb^−/−^)	MHCI mediated link between developmental synapse pruning and balanced synaptic learning rules enabling both LTD and LTP; direct requirement for MHCI molecule H2-Db in functional and structural synapse pruning in CNS neurons	MHCI molecule H2-Db is necessary and sufficient for synapse elimination in the retinogeniculate system
Moyer et al., 2016 [193]	In vivo preclinical study	Auditory Cortex	WT and kalirin KO^−/−^ mice	Decrease in numbers of spines between early adolescence and young adulthood Decrease in within-bouton GAD65 protein and GAD65-expressing bouton numbers between late adolescence and young adulthood	Relationship between structural changes of excitatory and inhibitory synapses during adolescence development and functional changes in auditory cortex relevant for the pathophysiology of schizophrenia
Yeung et al.; 2018	In vivo preclinical study	ACC; HC; DG	WT, HT Gabrb2^+/−^ and KO Gabrb2^+/−^ mice	Increasing synaptic transmission improved interneuron survival despite enhanced protein oxidation	The neuroinflammation was accompanied by elevated brain levels of oxidative stress marker MDA and the pro-inflammatory cytokines TNF-α and IL-6
Hasam-Henderson et al.; 2018	In vitro preclinical study	HC	N/A	NMDAR hypofunction and redox imbalance of the GSH system alter the maturation of the neuronal network activity and early life NMDAR hypofunction induces oxidative stress in interneurons, leading to decreased PV and GAD67 expression	Oxidative stress induced by NMDAR hypofunction and decreased GSH synthesizing capacity have been shown to lead to loss of parvalbumin

Several preclinical studies underline that the emergence of psychiatric symptoms in maternal inflammation models (i.e., late MIA) is mediated by PSD disorganization [202, 203] and changes in mRNA and protein levels of post-synaptic elements involved in plasticity and transmission.

For instance, neuroinflammatory conditions reproduced by an in vitro study through the exposure of hippocampal cultures to IL-1β, determined a 60% reduction in the expression of Arc, an immediate early gene product regulating the synaptic strength [204]. IL-1β exposure also resulted in a comparable reduction in Homer1a expression, an inducible key molecule of PSD participating in spine remodeling [204, 205]. Noteworthy, a similar suppression of Arc and Homer1a expression in the CA1 region of the hippocampus has been observed also in pharmacological models of schizophrenia not based on inflammation (i.e., those achieved by NMDAR antagonist administration [206]. Furthermore, animal models of brain inflammation obtained by LPS chronic administration exhibited increased cortical expression of constitutive Homer, Homer 1b/c. The increase in Homer1b/c, which competes with Homer1a at the PSD, appears to promote neuronal apoptosis via Bac-Bcl2 pathway, sustaining the progression of neuronal damage [207]. Pubertal LPS administration affects also PSD-95 protein expression in hippocampal regions but not in PFC, hypothalamus, and cerebellum of male mice 1 week after treatment [208]. It could be coherent with major susceptibly of this region to pubertal challenge with LPS, based on neurogenesis capability, high level of expression of receptors for stress hormones, and anatomical and functional links with the central stress response system [209–211]. Moreover, a significant decrease in c-Fos immunoreactive cells in the dentate gyrus has been reported in MIA offspring [212], potentially responsible for disruption in social behavior [213–215]. Early life insults may determine a decrease in PSD-95 in both the dorsal CA and dentate gyrus of the hippocampus, as well as a reduction in synaptic Ras GTPase-activating protein [186, 187].

Noteworthy, susceptible genotypes such as a mutation in Disrupted in schizophrenia 1 (*DISC1*), a hub protein particularly enriched in the PSD, may interact with prenatal inflammatory events, being responsible for a reduction of synaptic plasticity, impairment in neurotransmission, and widespread alterations in brain morphology [202]. Furthermore, a specific point mutation of the *DISC1* gene (L100P) interacts with MIA during gestation, resulting in an exacerbated phenotype of schizophrenia probably mediated by IL-6 [216]. Thus, even when not the “first hit,” inflammatory events may have a cumulative effect on individual genetic vulnerability.

In summary, inflammation represents one of the most relevant stressors in the structuring of PSD, as suggested by in vitro and animal modeling, reproducing PSD dysfunctions mimicking those observed in schizophrenia. It is through the extensive disorganization of PSD that many inflammatory cues may result in the subsequent impairment of the adaptive structural plasticity of dendritic spines.

methylated cytosines; *5hmC*, 5-hydroxymethylated cytosines; *mPFC*, medial prefrontal cortex; *GAD67*, 67-kDa isoform of GABA-synthesizing enzyme glutamic acid decarboxylase; *GAD65*, 65-kDa GAD isoform of GABA-synthesizing enzyme glutamic acid decarboxylase; *GAD1*, glutamate decarboxylase 1; *GAD2*, glutamate decarboxylase 2; *MeCP2*, methyl CpG-binding protein 2; *RGCs*, retinal ganglion cells; *LGN*, postsynaptic lateral geniculate nucleus; *LTD*, long-term depression; *H2-Db H2-Db*, MHCI molecule H2-Db monoclonal antibody; *CNS*, central nervous system; *WT*, wild type; *KO*, knock out; *GABBR2*, gamma-aminobutyric acid type B receptor subunit; *HT*, heterozygous; *ACC*, anterior cingulate cortex; *HC*, hippocampus; *DG*, dentate gyrus; *TNF-α*, tumor necrosis factor-alpha; *MDA*, malondialdehyde; *NMDAR*, N-methyl-D-aspartate receptor; *GSH*, glutathione; *N/A*, not applicable.

### The Effects of Inflammation on Astroglial Cells and Its Relevance for Synaptic Maturation

Within the functional unit of the tripartite synapse, astrocytes play a key role as active partners in the synaptic network, exchanging information with neuronal elements and regulating the transfer, processing, and storage of neural inputs, thus controlling synaptic plasticity in multiple neighboring neurons [221]. Inflammatory events may affect astroglial integrity and the astrocyte-neuronal coupling, jeopardizing the coordinated activity between neurons.

Of interest, an alteration in astrocyte arborization and survival has been demonstrated in the hippocampus of mice exposed to LPS in the prenatal period. An increase in both IL-1 and TNF-α has been shown after exposure to LPS with subsequent activation of microglia, promoting NO production via the p38MAPK/iNOS-dependent pathway, resulting in the opening of connexin 43 hemichannels [107]. The activation of connexin 43 hemichannels localized in astroglial cells of hippocampus favors the increase in intracellular Ca^2+^ via inositol-3-phosphate pathway and the release of glutamate [107]. Therefore, an imbalance of astroglial activity may contribute to the glutamate-induced excitotoxicity in schizophrenia animal models. Furthermore, IDO, a key enzyme that catalyzes the first step of the TRP/KYN pathway, is specifically located in astrocytes, not in microglial or other CNS cells. As previously described, immune responses modulate IDO activity through the release of cytokines such as IFN-γ or TNF-α, resulting in the accumulation of KYNA, which is detrimental for the glutamatergic system [118].

However, the astrocyte-neuronal coupling appears to be bidirectional. Hence, inflammatory-induced neuronal abnormalities, such as PSD alterations in glutamatergic synapses, may exert detrimental effects on the astrocytic phenotype, leading to change in morphology, size, and secretory profile. For example, Homer1, a scaffolding protein of PSD, has been observed to be downregulated during oxidative stress and neuroinflammation [222]. Homer1 suppresses the A1 deleterious astrocytic phenotype and promotes the conversion to A2 astrocytes, which are neuroprotective by producing anti-inflammatory cytokines and neurotrophic factors [217]. Therefore, the abnormalities in PSD induced by inflammatory conditions may alter the phenotypic polarization of neighboring astrocytes, which in turn, not providing support to neuron clusters, leads to a feedback loop that reverberates on the synapse.

The view of an overactivation of astrocytes in schizophrenia is also supported by clinical studies. For instance, S100B, a marker of astrocyte activation, has been found increased in serum and CSF of schizophrenia patients [223, 224].

Overall, these results suggest that inflammatory-based dysfunctions of astroglial cells contributes to the synaptic alterations and take part in schizophrenia pathophysiology.

### The Impact of Inflammation on Multiple Neurotransmitter Systems: Focus on Dopamine-Glutamate Interplay

Evidence is provided that inflammatory processes, together with immune responses, may trigger a network of changes in multiple neurotransmitter systems that underlie schizophrenia dysconnectivity and poor functional integration between and within different brain regions.

After mild maternal inflammation, mediated by a transient elevation of maternal blood/placental tryptophan levels and subsequent increase in tryptophan hydroxylase (TPH1) activity, an increase in 5-hydroxytryptamine (5-HT) receptor levels and overall abnormal formation of axonal circuitry of the serotonergic system has been reported [225–227]. Tryptophan metabolism is strongly influenced by inflammatory mechanisms in patients with schizophrenia and may take part in brain volume loss and attention impairment. Increased production of KYNA as a metabolite of tryptophan degradation pathway, consistent with inflammatory processes, resulted in NMDAR blockade in the PFC and dorsolateral prefrontal cortex (DLPFC) [228]. In fact, KYNA acts as an antagonist at this site, reproducing the NMDAR hypofunction characteristic of schizophrenia. It follows that serotonergic abnormalities induced by inflammatory events may converge on secondary glutamatergic dysregulations.

Glutamatergic transmission and synaptic plasticity may be modulated by discrete proteins which are essential for adaptive immunity. For instance, MHC-I complex influences the insertion on cell surface of the AMPAR involved in the induction of long-term depression (LTD) and the elimination of synapses [218, 220].

Neuroinflammatory responses in schizophrenia have also a relevant role in the definition of abnormalities in the GABA system. For instance, prenatal infections may be responsible for a reduction of the GABA-synthesizing enzyme activity, due to glutamic acid decarboxylase (GAD)-1 and GAD-2 promoter hypermethylation. In addition, the activity of GAD-65 and GAD-67 is also decreased via oxidative stress, leading to a reduction in GABA synthesis and release linked to early life NMDAR hypofunction [219, 229, 230]. Consistent with these findings, other preclinical studies reported an association between the oxidative stress, induced by NMDAR hypofunction and reduction in glutathione, the loss of PV + interneurons, and aberrant γ-band oscillatory activity [231].

**Fig. 5:**
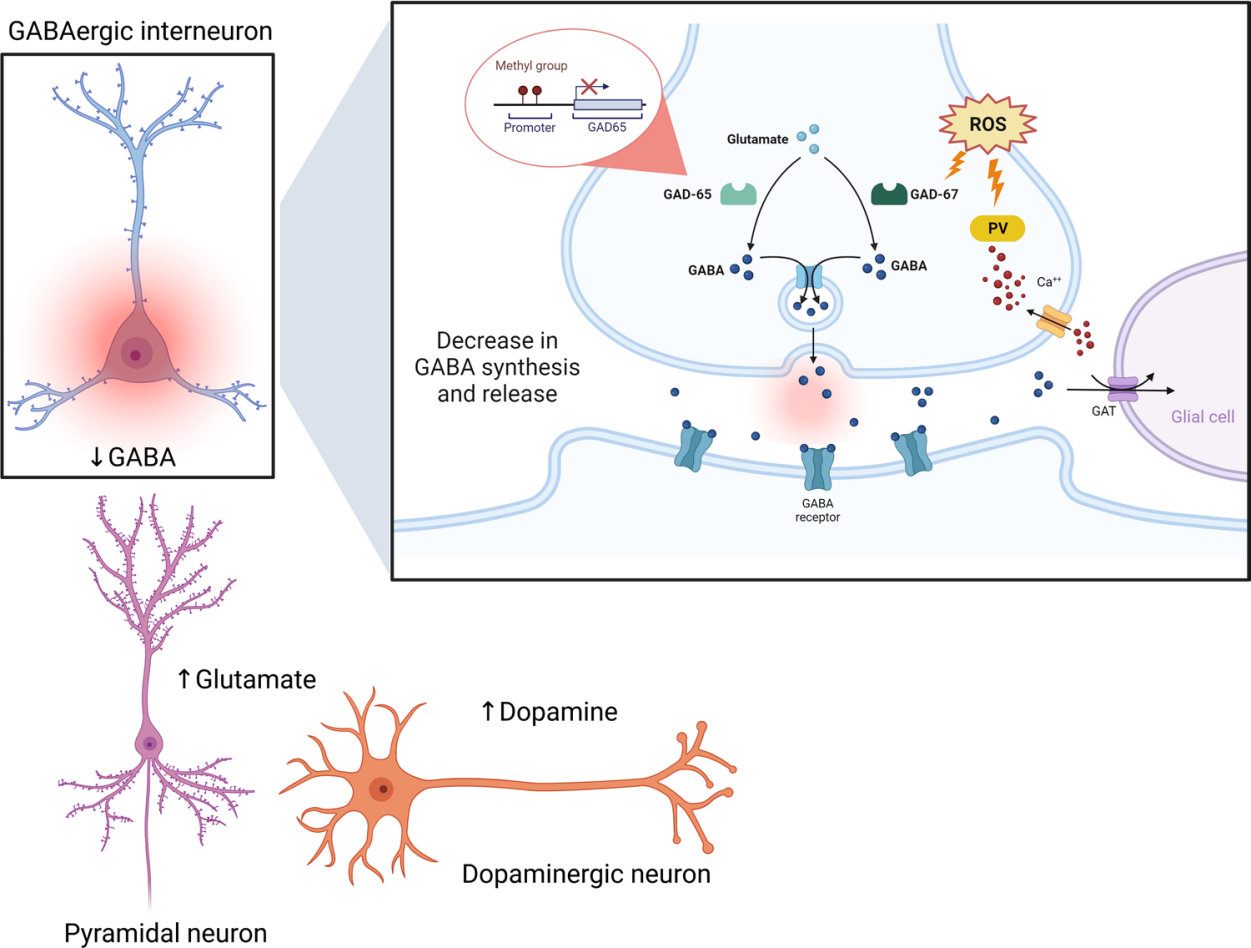
Neuroinflammatory responses in schizophrenia lead to abnormalities in the GABA system due to oxidative stress and hypermethylation in the promoter region of GABA-synthesizing enzymes. Reduced cortical GABA inhibition contributes, in turn, to overstimulation of downstream glutamatergic and dopaminergic neurons. GABA (γ-aminobutyric acid); GAD-65 (glutamic acid decarboxylase 65); GAD-67 (glutamic acid decarboxylase 67); ROS (reactive oxidative species); PV (parvalbumin); GAT (GABA transporter). Created with BioRender.com

Again, inflammatory-based abnormalities in the GABA transmission match those observed in the glutamatergic system, facilitating and contributing to excitotoxicity (Fig. 5).

If on the one hand inflammation modulates the survival and function of GABAergic interneurons, on the other hand abnormalities primarily occurring in the GABA system can lead to inflammatory responses. In fact, KO mice for *GABRB2* (GABA type A receptor β 2 subunit) gene exhibit schizophrenia-like behavior and GABA interneurons dystrophy, astrocyte degeneration, and widespread microglia activation in frontotemporal corticolimbic regions, as well as an increase in ionized calcium-binding adaptor molecule 1 (Iba-1), a molecular biomarker of neuroinflammation [232]. As also shown in clinical studies, polymorphism of *GABRB2* would confer not only susceptibility to the disorder but also resistance to antipsychotic treatment [233], probably due to the involvement of profound inflammatory alterations.

MIA affects also the pattern of expression of dopamine receptors, leading to increased dopamine levels and turnover in the striatum with a concomitant D2R-like decrease in adult offspring. The dopaminergic dysregulation in MIA offspring was accompanied by a behavioral phenotype reminiscent of TRS, characterized by the cognitive impairment being responsive to clozapine but not to haloperidol [174]. The increase in dopamine release is consistent with the upstream glutamatergic abnormalities described above and may represent the outcome of an altered glutamate-dopamine interplay due to inflammatory triggers.

In summary, several neurotransmitter systems are affected by early inflammatory insults, but most of these alterations a appear to converge on defects of glutamatergic synapses, resulting in disruption of synaptic plasticity and abnormal regulation of downstream dopaminergic circuits.

## Genetic Findings

Several studies suggested a correlation between inflammation genes and psychiatric disorders, showing that aberrant immune modulation increased the risk of developing schizophrenia; for instance, genome-wide-associated studies (GWAS) are very relevant to corroborate this hypothesis [234]. In fact, some of these GWAS have shown that the missense mutation of the Solute Family 39 Gene (*SLC39A8*), which encodes the Zinc Transporter Protein (Zrt), and the Zrt- and Irt-like Protein 8 (ZIP8), is associated with schizophrenia. Furthermore, ${\mathrm{ZIP}8}^{\mathrm{A}391\mathrm{T}}$’s loss of function results in downregulation of the synaptic glutamate receptors and dysregulation of NMDAR and AMPAR at the level of the GluN2A- and GluA1- subunits, respectively, as well as an alteration in the cytokine pathways of IL-6 and IL-10 [235]. To support the hypothesis of alteration of cytokine inflammatory homeostasis, several studies have been conducted. Furthermore, such abnormalities in cytokine cascades imply changes in synaptic stability. In this regard, Xiu et al. have shown that low levels of cytokine IL-10 have significant implications for schizophrenia, especially for the attentional performance which is significantly decreased by *IL-10–592 A/C* polymorphism. Therefore, it can be inferred that IL-10 and other anti-inflammatory cytokines can prevent neuronal dysfunction. Furthermore, *IL-10RA* and *IL-10RB* co-expressed genes have also been shown to promote changes during the synaptic remodeling process and synaptogenesis [236]. In addition, the polymorphism of the IL-1 receptor antagonist (*IL-1RN*) was correlated with an improved antipsychotic response in the negative symptom domain [237]. Moreover, Lang et al. refer to several genes that lead to immune signaling deficits by correlating them with schizophrenia, such as *DISC1* scaffold protein, regulator of G protein signaling 4 (*RGS4*), proline dehydrogenase 1 (*PRODH*), DiGeorge Syndrome critical region gene 2, and 6 (*DGCR2* and *DGCR6*) and zinc finger DHHC-type palmitoyltransferase 8 (*ZDHHC8*), V-akt murine thymoma viral oncogene homologue (*Akt)*, cAMP response element-binding protein (*CREB*), *IL-1B*, *IL-1RN*, and *IL-10* [238].

Schmitt et al. hypothesized that the synaptic functions in the left superior temporal cortex could be dysregulated by changes in the expression of genes involved in the immune process correlating with an increased risk of developing schizophrenia. Therefore, according to the authors, the genes that appear to correlate with the pathogenesis of schizophrenia are as follows: Chemokine Ligand 2 (*CCL2*), Chemokine Receptor 1 (*CCL1*), Endothelial Differentiation Sphingolipid G Protein–Coupled Receptor 3 (*EDG3*), Glutathione Peroxidase 1 (*GPX1*), MHC-II DP α 1 (*HLA-DPA1*), MHC-II DR β 3 (*HLA-DRB3*), IFN-γ Inducible Protein 16 (*IFI16*), IFN-α Receptor 2 (*IFN-αR2*), *IL-17F*, *IL-1α*, *IL-1β*, Interleukin 1 Receptor Accessory Integrin α 1 (*ITGA1*), Lymphocyte Protein 1 (*L-plastin*), Lipoprotein Lipase (*LPL*), Synthase (*LTC4S*), Methylenetetrahydrofolate Dehydrogenase NADP^+^ Dependent 2, Methenyltetrahydrofolate Cyclohydrolase (*MTHFD2*), Phospholipase A2 Group IIE (*PLA2G2E*), Prostaglandin E Receptor 4 (*PTGER4*), and Superoxide Dismutase 2 (*SOD2*) [239]. Calabrò et al. prove that single nucleotide polymorphism mutation (SNP) of MHC-I C (*HLA-C*) and MHC-DR α (*HLA-DRA*) could also be implied both in schizophrenia and inflammation [240].

Additionally, the results indicate an abnormal immune response in PV + neurons of the hippocampal areas of schizophrenia patients, which may be related to the findings of cognitive deficits in schizophrenia [241]. In addition, the following genes seem to play a pivotal role: Transglutaminase 2 (*TGM2*), POU Class 2 Homeobox 2 (*POU2F2*), Dual Specificity Phosphatase 1 (*DUSP1*), Proto-Oncogene Spi-1 (*SPI1*), SRY-Box Transcription Factor 11 (*SOX11*), IFN-γ (*IFNG*), Tripartite Motif-Containing 37 (*TRIM37*), Platelet-Derived Growth Factor Subunit B (*PDGF BB*), and Calpain 3 (*CAPN3*) [44].

Some evidence shows that there is an association between schizophrenia and allele A or G of TNF-α − 308 G/A [242] as well as Hasan et al. demonstrated that TNF-α − 238 G/A and − 308 G/A polymorphisms are not associated with schizophrenia but TNF-α − 238 G/A polymorphism may be related to treatment resistance and attempted suicide in patients with schizophrenia. In particular, the patients that carry the GG genotype of TNF-α − 238 G/A seem to have a higher risk of treatment resistance [243].

In conclusion, several pieces of evidence have suggested that dysregulation of immune genes could have a role in the pathogenesis of schizophrenia (Table 5), probably due to interference of normal neural signaling and alterations in dopamine-glutamatergic pathways.

**Table 5:** Genes linking inflammation to schizophrenia; the chromosome location was identified through the Ensembl Archive site (www.ensembl.org). TGM2, Transglutaminase 2; POU2F2, POU Class 2 Homeobox 2; DUSP1, Dual Specificity Phosphatase 1; SPI1, Proto-Oncogene Spi-1; SOX11, SRY-Box Transcription Factor 11; IFNG, Interferon γ; TRIM37, Tripartite Motif Containing 37; PDGF BB, Platelet-Derived Growth Factor Subunit B; CAPN3, Calpain 3; HLA-C, Major Histocompatibility Complex I-C; HLA-DRA, Major Histocompatibility Complex DR α; CCL2, Chemokine (C–C motif) Ligand 2; CCL1, Chemokine (C–C motif) Receptor 1; EDG3, Endothelial Differentiation, Sphingolipid G-Protein-Coupled Receptor 3; GPX1, Glutathione Peroxidase 1; HLA-DPA1, Major Histocompatibility Complex II DP α 1; HLA-DRB3, Major Histocompatibility Complex II DR β 3; IFI16, Interferon, γ-Inducible Protein 16; IFNAR2, Interferon α Receptor 2; IL-17F, Interleukin 17F; IL-1A, Interleukin 1 α; IL-1B, Interleukin 1 β; ITGA1, Integrin α; LCP1/L-plastin, Lymphocyte Cytosolic Protein 1; LPL, Lipoprotein Lipase; LTC4S, Leukotriene C4 Synthase; MTHFD2, Methylenetetrahydrofolate Dehydrogenase NADP+ Dependent 2, Methenyltetrahydrofolate Cyclohydrolase; PLA2G2E, Phospholipase A2 Group IIE; PTGER4, Prostaglandin E Receptor 4; SOD2, Superoxide Dismutase 2; IL-10, Interleukin 10; IL-10RA, Interleukin 10 receptor subunit α; IL-10RB, Interleukin 10 receptor subunit β

Authors	Study design	Subjects	Gene symbol	Chromosomal location
Gatta et al., 2021 [44]	Cross-sectional	Nonpsychotic controls (*n* = 18) Individuals with schizophrenia (*n* = 19)	***TGM2***	20q11.23
			***POU2F2***	19q13.2
			***DUSP1***	5q35.1
			***SPI1***	11p11.2
			***SOX11***	2p25.2
			***IFNG***	12q15
			***TRIM37***	17q22
			***PDGF BB***	22q13.1
			***CAPN3***	15q15.1
Calabrò et al., 2015 [240]	Cross-sectional	Nonpsychotic controls (*n* = 4477) Individuals with schizophrenia (*n* = 4486)	***HLA-C***	6p21.33
			***HLA-DRA***	6p21.32
Schmitt et al., 2011 [239]	*Post-mortem*	Nonpsychotic controls (*n* = 10) Individuals with schizophrenia (*n* = 10)	***CCL2***	17q12
			***CCL1***	17q11.2-q12
			***EDG3***	9q22.1
			***GPX1***	3p21.31
			***HLA-DPA1***	6p21.32
			***HLA-DRB3***	6p 21.31
			***IFI16***	1q23.1
			***IFNAR2***	21q22.11
			***IL-17F***	6p12.2
			***IL-1A***	2q14.1
			***IL-1B***	2q14.1
			***ITGA1***	5q11.2
			***LCP1/*** ***L-plastin***	13q14.13
			***LPL***	8p21.3
			***LTC4S***	5q35.3
			***MTHFD2***	2p13.1
			***PLA2G2E***	1p36.13
			***PTGER4***	5p13.1
			***SOD2***	6q25.3
Xiu et al., 2016 [236]	Cross-sectional	Nonpsychotic controls (*n* = 256) Individuals with schizophrenia (*n* = 540)	***IL-10***	1q32.1 **(mutation 592 A/C)**
			***IL-10RA***	11q23.3
			***IL-10RB***	21q22.11

## Implications for Treatment: the Effect of Antipsychotics on Inflammatory Markers in Schizophrenia

The role of inflammation in the pathophysiology of schizophrenia has been suggested by several findings of the therapeutic benefit of anti-inflammatory drugs in schizophrenia. Some authors have suggested that the impact of atypical antipsychotics on peripheral inflammatory mediators in schizophrenia patients could improve the search for new therapeutic strategies by correlating them with clinical response through modulation of proteins that play a role in inflammation and immune system pathways [244].

Previous studies have shown that antipsychotic treatment may be associated with suppressing pro-inflammatory cytokine levels and increasing anti-inflammatory cytokines (Table 6). The effect of antipsychotic treatment on cytokine levels has been reported by a meta-analysis that found a significant reduction in IL-1β, IL-2, and IL-6 after antipsychotic treatment in drug-naïve patients with first-episode psychosis (FEP), while TNF-α, IL-17, and IFN-γ were still elevated, suggesting that they might serve as markers for psychosis [50]. Another meta-analytic study found an elevation in sIL-2R and IL-12, with IL-1β, IL-2, and IL-6 reduced, after a mean period of 53 days of antipsychotic treatment in patients with FEP and chronic schizophrenia [48, 50, 245, 246].

**Table 6:** The effect of antipsychotics on inflammatory markers in schizophrenia and schizophrenia animal models. SCZ, schizophrenia; UHPLC, ultra-high-pressure liquid chromatography; PBMC, peripheral blood mononuclear cell cultures; TRS, treatment -resistant schizophrenia; non-TRS, non-treatment-resistant schizophrenia; IRS, inflammatory response system; ELISA, enzyme-linked immunosorbent assay; FEP, first episode schizophrenia; CRP, C-reactive protein; IL, interleukin; ABTS + , histopaque, 3-ethylbenzo-thiazoline-6-sulfonic acid (2,20-azinobis); DPPH, 2,2-diphenyl-1-picrylhydrazyl; PHA, phytohemagglutinin; LPS, lipopolysaccharide; PCP, phencyclidine; ROS, reactive oxygen species; NO, nitric oxide; CCI, cecal content injection; D1R, dopamine receptor 1; TNF, tumor necrosis factor; sTNFr, soluble tumor necrosis factor receptor; IFN, interferon; CC16, 16 kDa Clara cell protein; sIL-2r, soluble interleukin 2 receptor; sIL-6R, soluble interleukin 6 receptor; TGF, transforming growth factor; NF-κB, nuclear factor kappa B; TLR-4, toll-like receptor 4; CD80, monoclonal mouse anti-human antibodies; COX-2, cyclooxygenase 2; PANSS, positive and negative syndrome scale; iNOS, inducible nitric oxide synthase; poly(I:C), viral mimetic polyriboinosinic-polyribocytidilic acid; Iba-1, microglia/macrophage-specific calcium-binding protein; EP, early psychosis; Bcl-2, anti-apoptotic marker; LC3-II, autophagosome marker 1A/1B-light chain 3, NSC, neural stem cells, SVZ, subventricular zone; N/A, not applicable; PFC, prefrontal cortex

Clinical/preclinical studies	Authors	Study design	Methodology/samples	Subjects/models	Antipsychotics	Outcome
Clinical studies	Garcia-Rosa et al., 2020 [244]	Longitudinal study (6 weeks)	Plasma proteome using 2D-UPLC-HDMSE	Early-stage SCZ patients (*n* = 26)	Olanzapine (*n* = 11); risperidone (*n* = 15)	Modulation of proteins that play a role in inflammation and/or immune system pathways
	Pollmächer et al., 1996 [282]	Longitudinal study (6 weeks)	Enzyme-linked immunosorbent assays in plasma	SCZ patients (*n* = 27)	Clozapine (*n* = 27)	↑TNF-α, sTNFr (p55 and p75), sIL-2r
	Müller et al., 2004 [268]	RCT (5 weeks)	ELISA in serum	SCZ patients (*n* = 50)	Risperidone + placebo (*n* = 25); risperidone + celecoxib (*n* = 25)	The cytokines and lymphocytes reflected the type-1/type-2 balancing effects of COX-2 inhibitors, celecoxib add-on therapy group showed a significant group effect in the PANSS total score
	Lin et al., 1998 [24]	Cross-sectional	ELISA in plasma	TRS patients (*n* = 15); non-TRS patients (*n* = 12); healthy controls (*n* = 15)	Haloperidol (*n* = 27)	↑IL-6, sIL-6R; ↓CC16
	Maes et al., 2000 [248]	Longitudinal study (4 months)	ELISA in plasma	TRS patients (*n* = 17); non-TRS patients (*n* = 14); healthy control (*n* = 7)	TRS: clozapine (*n* = 12); risperidone (*n* = 5) Non-TRS: haloperidol (*n* = 5); clozapine (*n* = 4); risperidone (*n* = 4); perphenazine (*n* = 1)	TRS and non-TRS = ↑IL-6, ↓CC16; TRS = ↑sIL-6R
	Steiner et al., 2020 [249]	Longitudinal study (6 weeks)	Multiplex immunoassay in serum	FEP patients (*n* = 129); non-FEP patients (*n* = 124); healthy controls (*n* = 294)	Olanzapine (*n* = 71); quetiapine (*n* = 17); risperidone (*n* = 45); aripiprazole (*n* = 15); typical antipsychotics (*n* = 9); other drugs/combinations (*n* = 6)	At the baseline: ↑Neutrophils, monocytes, CRP, ↓Eosinophils; Neutrophils, eosinophils, and CRP partially remitted after antipsychotic treatment
Preclinical studies	Brinholi et al., 2016 [251]	In vitro	Chemiluminescence in neutrophils from whole blood	SCZ patients (*n* = not retrieved)	Clozapine; olanzapine; quetiapine; risperidone; ziprasidone	Clozapine and olanzapine have antioxidant effects by scavenging superoxide anion on the respiratory burst; ziprasidone significantly scavenged ABTS + and stabilized the radical DPPH; risperidone significantly reduced the respiratory burst; haloperidol and quetiapine lacked antioxidant effects
	Song et al., 2000 [253]	In vitro	Whole blood stimulated by PHA + LPS	Healthy volunteers (*n* = 9)	Clozapine haloperidol	↑IL-1RA
	Gross et al., 2003 [255]	In vitro	Peripheral blood monocytes performed with a counter coulter	SCZ patients showing unsatisfactory response to treatment (*n* = 8)	Clozapine	↓ROS
	Al-Amin et al., 2013 [245]	In vitro	Enzyme-linked immunosorbent assays in PBMCs stimulated by LPS and poly(I:C)	FEP patients (*n* = 12)	Haloperidol; quetiapine; clozapine; risperidone	Haloperidol, quetiapine, clozapine = ↑IL-4, IL-10, ↓IFN-γ; risperidone = ↑IL-10, ↓IFN-γ
	Hu et al., 2012 [256]	In vitro	Primary cortical and mesencephalic neuron-glia cultures pretreated by clozapine and exposed to LPS	Mice and rats	Clozapine	↓Microglia-derived superoxide, intracellular ROS, NO, and TNF-α
	Park et al., 2019 [259]	In vitro	ELISA in dendritic cells treated simultaneously with LPS and trifluoperazine	LPS and cecal CCI induced endotoxemia in mice	Trifluoperazine	↓TNF-α, IL-6, IL-10, TGF-β
	Yamamoto et al., 2016 [261]	In vitro	ELISA in RAW 264 cells and in primary macrophages exposed to LPS	Mice	Haloperidol	↓NF-κB activation, expression of CD80, IL-1β, IL-6, IL-12 p40
	Ribeiro et al., 2013 [283]	In vitro	Immunofluorescence staining for Iba-1 in brain slices	Early immune activation with poly(I:C) in rats	Clozapine	↓Microglial activation, iNOS
	Lundberg et al., 2020 [284]	In vitro	Western blot and mitochondrial DNA analysis	Mice NSCs exposed to ketamine	Clozapine	↑Bcl-2; ↓pro-apoptotic cleaved form of caspase-3, LC3-II
	Paterson et al., 2006 [254]	In vitro	Immunocytochemistry in brain PFC slices	Rats after acute and chronic PCP administration	Clozapine haloperidol	↓TNF-α

The 16 kDa Clara cell protein (CC16) is an endogenous anti-inflammatory protein with immunosuppressive and anti-inflammatory effects related to IRS activation. Furthermore, CC16 may inhibit the biological activity of IFN-γ, produced by stimulated leukocytes, and IL-6. Low serum concentrations of CC16 have been found accompanied by increased plasma concentrations of IL-6 and IL-6R (IL-6 receptor) in schizophrenia patients. Therefore, lower concentrations of CC16 in schizophrenia may be causally related to IRS activation, as indicated by increased serum concentrations of IL-6 and IL-6R. Significantly higher serum levels of IL-6 were found in TRS patients compared to healthy controls, while those of non-TRS did not differ in serum levels of IL-6 from controls [24]. These results support the hypothesis that IRS activation is involved in the pathophysiology of schizophrenia and is related to resistance to antipsychotic agents [24, 247, 248].

Furthermore, several authors have been suggested a relationship between the atypical characteristic of antipsychotics, such as olanzapine and clozapine, and the ability to exert anti-inflammatory and antioxidant effects [249–252]. Since in vitro and in vivo studies have reported that clozapine modulates the production of serum inflammatory cytokines such as IL-6 and IFN-γ [253, 254] whereas clozapine in vivo reduces ROS production by monocytes from schizophrenia patients [255], Hu et al. postulated that clozapine has neuroprotective properties on dopaminergic neurons counteracting neurodegeneration triggered by inflammation [256].

Howes et al. propose that immune-synaptic interactions could be targeted by therapeutic approaches, based on evidence of multiple genetic risk factors related to the immune system, increased microglial cell density, and decreased synaptic terminal density in schizophrenia [14]. Several authors using in vivo PET imaging of synaptic vesicle glycoprotein 2a (SV2A) detectable in the synaptic terminals, showed significantly lower levels of SV2A in FC and anterior cingulate cortex (ACC) in schizophrenia, whereas exposure to antipsychotic drugs does not significantly alter SV2A levels or specific binding in the FC and cingulate cortices of naïve rats [257]. These findings suggested that synaptic alterations may occur in vivo in schizophrenia and that lower levels of SV2A are unlikely to be directly accounted for by antipsychotic drug treatment in schizophrenia [257].

Preclinical studies have demonstrated the interaction between the dopaminergic system and the immune system displaying that antipsychotic drugs act as a regulators of inflammation. Specifically, the antipsychotic trifluoperazine suppresses dopamine secretion, regulates pro-inflammatory cytokines, and increases survival rates in animal models of inflammation. Another finding demonstrating the role of antipsychotic drugs in inflammation is the inhibitory action of paliperidone on TLR-4 in a stress-induced neuroinflammation model in rats. In line with these studies, some authors showed that haloperidol attenuated nuclear factor kappa B (NF-κB), a transcription factor involved in both immune system and cognitive functioning regulation [258], and consequently blocked the production of pro-inflammatory cytokines in response to LPS. Taken together, these findings demonstrate that antipsychotic drugs used to treat schizophrenia may induce anti-inflammatory effects by acting on the dopaminergic system and immune cells, according to the hypothesis on the critical role of dopamine in regulating inflammation [116, 259–262].

In contrast, other authors have suggested that the dysregulation of the immune response associated with schizophrenia is a consequence of disease progression or due to long-term treatment with antipsychotic drugs [263, 264]. Evidence from animal models, post-mortem human brain, and peripheral blood of schizophrenia patients identified an increase in acute-phase response signaling proteins, such as α-1 antichymotrypsin, a cathepsin G inhibitor released at the site of inflammation. A recent study of the plasma proteome of patients with schizophrenia showed a decrease in this protein after the use of atypical antipsychotics. Therefore, on one hand, antipsychotic treatment can reduce markers of inflammation; on the other hand, add-on anti-inflammatory agents may be helpful in mitigating psychotic symptoms [188, 265–270].

An immunological understanding of schizophrenia could be clinically convenient because inflammation is associated with poor antipsychotic response, suggesting that measuring inflammation levels as part of the routine clinical assessment of psychosis could identify treatable causes of inflammation and potentially guide antipsychotic treatment decisions [271, 272]. Ketamine blockade of NMDAR leads to hypofunction of this receptor and is linked to schizophrenia, producing positive symptoms of psychosis, but its indirect action on the dopaminergic system also induces negative and cognitive symptoms associated with schizophrenia [272, 273]. Preclinical evidence has investigated the acute effect of ketamine treatment in peripheral blood, serum, and brain tissue in rats, identifying alterations associated with inflammation and growth factor signaling. In particular, serum multiplex immunoassay profiling identified altered levels of IL-4, TNF-α, and fibroblast growth factor-9 (FGF9), suggesting a link between ketamine exposure and peripheral inflammation and dysregulation of growth factors. In addition, mass spectrometry profiling of rat brain tissue found proteomic changes in the FC and to a greater extent in the hippocampus, involving modifications in signaling kinases and proteases included Ca^2+^ calmodulin dependent protein kinases (CamKII), extracellular signal-regulated kinase 1 (ERK1), mTOR, and protein kinase C β type (PKCβ), all considered to be ketamine-induced effects on signaling pathways involved in synaptic plasticity [274]. Specifically, the overactivity of PKCβ signaling, implied in inflammatory processes [275], may cause alterations in PFC regulation of working memory [276], identified as a central dysfunction in schizophrenia [277]. Multiple lines of evidence have suggested that PKCβ modulates D2 autoreceptor–activated transporter trafficking and dopaminergic signaling [278]. Furthermore, PKCβ abnormalities have been implicated in disorders related to abnormal dopamine extracellular levels, such as schizophrenia [278]. In addition, in vitro and in vivo data have reported a potential role for PKCβ in weight gain induced by antipsychotic treatment [279]. Regulation of PKCβ may represent a potential pharmacological target for regulating abnormal extracellular dopamine levels and preventing metabolic side effects of chronic therapy with antipsychotics, such as clozapine [274, 276–280]. The atypical antipsychotic clozapine, with a peculiar receptor profile, has been shown to affect multiple neurotransmitter systems, including NMDA receptor agonism [281], and possibly also impacts the immune system [282].

### Anti-inflammatory Treatments Modulate Synaptic Plasticity in Schizophrenia

Given the potential anti-inflammatory action of antipsychotics, it has been proposed that immunomodulatory strategies may contribute to improving psychotic symptoms of schizophrenia. GWAS have indicated a close relationship between the immune system and schizophrenia, opening research to new therapeutic strategies, such as the application of human umbilical cord–derived mesenchymal stem cells (hUC-MSC) associated with an immunomodulatory effect. In fact, in amphetamine-sensitized mice showing neuroinflammation, peripheral increase in TNF-α, and schizophrenia-like behavior, a single infusion of hUC-MSC could have a long-term beneficial effect through induction of Treg and secretion of IL-10 [285].

Celecoxib, a COX-2 inhibitor, was tested in patients affected by schizophrenia during acute exacerbations as augmentation therapy of risperidone. In this study, celecoxib add-on therapy compared resulted to be superior than risperidone alone [286].

Several studies showed beneficial clinical effects of COX-2 inhibition on cognition in the early stages of the disease, in contrast to others that found no relevant benefit in chronic schizophrenia. In addition, a study of celecoxib in add-on to amisulpride demonstrated a beneficial effect at the onset of schizophrenia on both positive and negative symptoms [286–290]. COX-2 inhibition could be a promising approach in schizophrenia therapy, as it can balance the type1/type2 immune response, reduce astrocytes activation, and decrease levels of the NMDAR antagonist KYNA [291] through inhibition of IL-6 and prostaglandin E2 (PGE2) [292].

Clinical studies in patients with schizophrenia treated with combinatorial use of anti-inflammatory and antipsychotic agents during the early stages of schizophrenia have shown promising beneficial effects by improving negative and cognitive symptoms compared to patients receiving antipsychotic drugs alone [290, 293]. Further evidence showed that effective anti-inflammatory agents in schizophrenia patients were aspirin, minocycline, and N-acetylcysteine (NAC), reducing the severity of symptoms in FEP or early stage of schizophrenia [269, 270, 294]. A meta-analysis of the clinical effects of nonsteroidal anti-inflammatory drugs (NSAIDs) revealed a significant improvement in patients with a short duration of the disease or in the FEP [295]. A wide range of pharmacological agents may modulate the function of microglia, which when activated play a role in inflammation associated with psychiatric disorders. Many existing non-psychiatric treatments such as statins, NSAIDs, NAC, minocycline, and natalizumab, as well as psychological interventions that address stress reactivity, could indirectly influence microglial function [283, 296–300].

Cysteamine and cystamine have been reported to mitigate inflammation and increase neuroprotective pathways, respectively, involving BDNF and related nuclear factor erythroid 2 (Nrf2) signaling, with a role in counteracting neurodegeneration and neuropsychiatric deficits [301]. It has been suggested that combination treatment using anti-inflammatory drugs with antipsychotics may attenuate psychotic symptoms by dampening the production of inflammatory cytokines, ROS, prostaglandins, and activated microglia. For example, it has been shown that treatment with the anti-inflammatory drug minocycline, an antibiotic and inhibitor of microglial activation, combined with risperidone, olanzapine, quetiapine, clozapine, or chlorpromazine, could alleviate negative symptoms [270, 293, 302]. The promising antipsychotic effects of minocycline has been supported by both preclinical and clinical studies [293, 303–305]. In addition, minocycline may reduce the aberrant synaptic pruning that occurs in the early stages of schizophrenia. Furthermore, 12 months of minocycline supplementation in early-onset schizophrenia appears to protect against gray matter loss in frontotemporal cortical regions [93, 302, 306]. Several authors proposed that pro-inflammatory cytokines released by activated microglia adversely affect the function of the dopaminergic and glutamatergic systems; therefore, it could be plausible to consider the restoration of resting microglia as a potential approach for treating schizophrenia [307, 308].

Schizophrenia research emphasizes the need for new therapeutic approaches based on antioxidant and anti-inflammatory compounds. A hallmark of schizophrenia is a dysfunction of PV + fast-spiking interneurons (PVI), which are essential for neuronal synchronization affecting sensory perception and cognition during early brain development [309–316]. Various mechanisms involved in schizophrenia pathophysiology, such as dopamine dysregulation, glutamate dysfunction, and neuroinflammation, appear to converge towards oxidative stress affecting PVI and their PNN. MMP9 is released from neurons, astrocytes, and microglia in the CNS and is modulated by cytokines, growth factors, and ROS in both normal and pathological conditions. It has been found that MMP9 is involved in a number of key neurodevelopmental processes, including the maturation of inhibitory neurons containing the calcium-binding protein parvalbumin, the formation of the specialized extracellular matrix structure of the PNN, myelination, and synaptic pruning, relevant for schizophrenia. Some authors suggested that MMP9 may modulate synaptic plasticity through the cholinergic, noradrenergic, and possibly also dopaminergic systems, controlling synaptogenesis and structural plasticity of dendritic spines. In this context, it has been hypothesized that dysregulation of MMP9 is related to the pathogenesis of schizophrenia [317, 318]. Oxidative stress–induced microglia and redox-sensitive MMP9 lead to the activation of receptor for advanced glycation end products (RAGE) in soluble and nuclear forms and then secretion of NF-κB. A preclinical study showed that the combination of antioxidant treatment and environmental enrichment applied during the adolescent and juvenile periods, respectively, normalizes the integrity and function of PVI/PNN in the ACC in mice. This recovery is mediated by NAC, possibly through inhibition of the oxidative stress–induced MMP9/RAGE pathway, conferring neuroprotection in an animal model with a genetic risk of impaired antioxidant defense. The addition of NAC to the antipsychotic treatment in early psychosis patients has been suggested to also rescue the MMP9/RAGE pathway, increase prefrontal GABA levels, and may improve clinical symptoms and cognitive function, confirming a similar neuroprotective action [317, 318].

Several authors have proposed that preventing dendritic spine loss in individuals at high risk of the disease during the prodromal or transient phase of psychosis could be a key goal in the treatment of schizophrenia [319–322]. In this context, the antipsychotic clozapine, the gold standard for TRS, has been shown to promote the formation of dendritic spines by increasing some of the PSD proteins, such as spinophilin or shank1a. Some authors reported the effect of clozapine to promote the growth of dendritic spines compared to haloperidol. Furthermore, in vitro studies found that clozapine could have a protective/anti-apoptotic effect on adult neural stem cells challenged with ketamine [284, 323]. It is plausible to speculate that clozapine protective effect on the dendritic spine is mediated by the anti-oxidant and anti-inflammatory effects of this molecule [251–256]. In view of the pronounced inflammatory substrate that has been observed in TRS, it is possible that clozapine is more effective than other antipsychotics in light of this additional anti-inflammatory property.

A future perspective is to determine which schizophrenia patients are likely to benefit from anti-inflammatory therapies through a concerted approach including immune target identification using genomic, deep immunophenotyping and other methods. In this context, further experimental clinical and animal studies examining the effects of novel immunomodulating agents on the brain and behavior are required.

## Discussion

In the last decade, multiple evidence from clinical, preclinical, and post-mortem studies have corroborated the inflammation and immune-related hypothesis of schizophrenia pathogenesis [3, 4, 324] fueling more robust interpretation on the role of inflammatory and immune processes in the framework of neurotransmitters and synaptic alterations of the disease: among all dopamine-glutamate interaction [116] and dendritic spine modification [5].

First, the effects of inflammation on dopamine function have been demonstrated, somewhat unexpectedly, being bidirectional [22]: while on one side inflammation influences dopamine release and possibly post-synaptic effects of the neurotransmitter, dopamine on the other side has been proven to have a significant involvement in modulating a few discrete mechanisms of inflammation in the CNS [112, 114].

Second, the link between inflammation and related immune processes is even more evident concerning glutamate in MIA models, where altered glutamate release and NMDAR expression in the PFC and hippocampus [142] suggested the involvement of glutamate storm in the process of neuroprogression [5]. Furthermore, preclinical evidence showed an inflammation/immunity-induced disruption of PSD scaffold proteins, crucial for downstream NMDAR signaling and synaptic efficacy, leading to the aberration of dopamine-glutamate interaction. It has been hypothesized that dopamine-glutamate interaction is the core synaptic and intracellular signaling disrupted in schizophrenia, according to in vivo PET evidence [12], in vitro and in vivo animal model studies [13], and post-mortem brain tissue analysis [14–18]. In addition, altered glutamatergic synaptic activity could impact the regulation of interneuron function and the balance between excitation and inhibition in cortical circuits, potentially leading to disinhibition of dopamine release control [90, 94, 95]. Dysfunction of GABAergic inhibitory interneurons could induce a glutamate storm from excitatory glutamatergic cortical pyramidal neurons and a hyperdopaminergic subcortical state [5].

The fact that inflammation and immune response may be strongly interconnected with dopamine and glutamate neurotransmission could be supported by the presentation of psychotic symptoms reminiscent of schizophrenia that may occur during CNS infections [19] and autoimmune disorders [20, 21] indicating a reciprocal interaction between neurotransmitter systems and immune mediators [22].

It has been found that neuroinflammation induced by overactive microglia can reduce the density of spines and synapses on glutamatergic cortical pyramidal neurons below a critical threshold for integrated network functioning, relevant to schizophrenia [91–93]. The glutamatergic hypothesis of schizophrenia, therefore, could be complemented by the contribution of the immune system to the etiology of the disease, explaining the excessive synaptic pruning that occurs during late adolescence or early adulthood in schizophrenia [5, 93, 325]. Several authors have proposed that pro-inflammatory cytokines released by activated microglia adversely affect the function of the dopaminergic and glutamatergic systems, so it may be plausible to consider restoring microglial function as a potential approach to treating schizophrenia [307, 308].

Third, a potential link between dopamine-glutamate interaction, synaptic changes and immune system is supported by recent findings showing significant increase in mRNA encoding complement receptors, regulators, and proteins in the plasma of schizophrenia patients compared to healthy controls. A rise of inflammatory indices obtained from mRNA expression patterns was found associated with reduced cortical thickness in schizophrenia patients. Therefore, the upregulation of the complement cascade significantly affected cortical integrity and functional connectivity, relevant for cognitive deficit characteristic of schizophrenia [142].

Fourth, the inflammatory/immune theory of schizophrenia molecular pathophysiology pose relevant questions for the therapy of the disease and specifically for those forms of the disease that are poor or not responsive to available therapies: i.e., TRS and ultra-TRS, the former being the canonical treatment resistance for which clozapine has indication and the latter the one that is not responsive even to clozapine. It has been postulated that immune system and inflammation involvement could influence the response to antipsychotic action. In line with this interpretation, genes related to the immune response have been detected as discriminant for the efficacy of antipsychotic treatment.

One recent and stringent example comes from the observations that common variation within *CSMD1*, which encodes a putative complement inhibitor, has consistently associated with schizophrenia at genome-wide significance [326].

Novel therapeutic approaches targeting aberrant neurodevelopment and disease progression have been explored, focusing on aberrant synaptic plasticity, glutamate storm, dendritic cell apoptosis, calcium channel dysfunction, and microglial activation, especially in the early stages of the disease. There is growing support for the hypothesis that preventing the loss of dendritic spines in high-risk individuals during the prodromal or transient phase of psychosis could be a potential target in the treatment of schizophrenia [319–322]. In this context, the antipsychotic clozapine, the only approved treatment for the TRS condition, has been shown to increase PSD proteins, favoring the formation of dendritic spines [284, 323].

It has been reported that antipsychotic drugs can reduce inflammatory effects by acting on the dopaminergic system and immune cells, following the thesis of dopamine as a regulator of inflammation [116, 259–262]. On the other hand, the glutamatergic hypothesis of schizophrenia may be linked to the immune system through the inflammation-induced susceptibility to developmental abnormalities described in the “two-hit model” of schizophrenia, in which genetic factors combine in a complex manner with environmental insults and concur in the development of the disease [128].

Regarding the role of the inflammatory process in schizophrenia, anti-inflammatory therapy in addition to antipsychotics needs further research [327]. COX-2 inhibition [128] or the use of the antibiotic minocycline [293, 305], among others, could be viable option. Therapeutic research, however, must consider different mechanisms for treatment targets in the neuroimmune system and dopaminergic-glutamatergic neurotransmission circuits.

In summary, the evidence supporting the involvement of inflammation and immune responses in the pathophysiology of schizophrenia have justified the need for further efforts to consider and interpret the most relevant findings for exploring significant novel strategies of treatment of schizophrenia and at same time reveal new crucial questions.

## Data Availability

The datasets generated and analyzed during the current study are available from the corresponding author on reasonable request.
